# CD8^+^ T Cells Involved in Metabolic Inflammation in Visceral Adipose Tissue and Liver of Transgenic Pigs

**DOI:** 10.3389/fimmu.2021.690069

**Published:** 2021-07-12

**Authors:** Kaiyi Zhang, Cong Tao, Jianping Xu, Jinxue Ruan, Jihan Xia, Wenjuan Zhu, Leilei Xin, Huaqiong Ye, Ning Xie, Boce Xia, Chenxiao Li, Tianwen Wu, Yanfang Wang, Martine Schroyen, Xinhua Xiao, Jiangao Fan, Shulin Yang

**Affiliations:** ^1^ State Key Laboratory of Animal Nutrition, Ministry of Agriculture Key Laboratory of Animal Genetics Breeding and Reproduction, Institute of Animal Science, Chinese Academy of Agricultural Sciences, Beijing, China; ^2^ Gembloux Agro-Bio Tech, University of Liège, Gembloux, Belgium; ^3^ The Ministry of Health Key Laboratory of Endocrinology, Department of Endocrinology, Peking Union Medical College Hospital, Peking Union Medical College, Chinese Academy of Medical Sciences, Beijing, China; ^4^ College of Animal Sciences & Technology, Huazhong Agricultural University, Wuhan, China; ^5^ Faculty of Medicine, University of Helsinki, Helsinki, Finland; ^6^ Shanghai Key Laboratory of Children’s Digestion and Nutrition, Department of Gastroenterology, Xinhua Hospital, Shanghai Jiaotong University School of Medicine, Shanghai, China

**Keywords:** pig model(s), metaflammation, CD8, T cells, liver inflammation, obesity, adipose tissue

## Abstract

Anti-inflammatory therapies have the potential to become an effective treatment for obesity-related diseases. However, the huge gap of immune system between human and rodent leads to limitations of drug discovery. This work aims at constructing a transgenic pig model with higher risk of metabolic diseases and outlining the immune responses at the early stage of metaflammation by transcriptomic strategy. We used CRISPR/Cas9 techniques to targeted knock-in three humanized disease risk genes, *GIPR^dn^*, *hIAPP* and *PNPLA3^I148M^*. Transgenic effect increased the risk of metabolic disorders. Triple-transgenic pigs with short-term diet intervention showed early symptoms of type 2 diabetes, including glucose intolerance, pancreatic lipid infiltration, islet hypertrophy, hepatic lobular inflammation and adipose tissue inflammation. Molecular pathways related to CD8^+^ T cell function were significantly activated in the liver and visceral adipose samples from triple-transgenic pigs, including antigen processing and presentation, T-cell receptor signaling, co-stimulation, cytotoxicity, and cytokine and chemokine secretion. The similar pro-inflammatory signaling in liver and visceral adipose tissue indicated that there might be a potential immune crosstalk between the two tissues. Moreover, genes that functionally related to liver antioxidant activity, mitochondrial function and extracellular matrix showed distinct expression between the two groups, indicating metabolic stress in transgenic pigs’ liver samples. We confirmed that triple-transgenic pigs had high coincidence with human metabolic diseases, especially in the scope of inflammatory signaling at early stage metaflammation. Taken together, this study provides a valuable large animal model for the clinical study of metaflammation and metabolic diseases.

## Introduction

Obesity-related diseases such as type 2 diabetes mellitus (T2DM), non-alcoholic fatty liver disease (NAFLD), and atherosclerosis are a cluster of chronic metabolic disorders with multifactorial etiology, involving genetic variation, western diet, and unhealthy lifestyle (1). In the past few decades, obesity and the associated diseases have become important public health problems worldwide, risking human life and bringing substantial financial burden. It is well established that the chronic, low-grade inflammation originated from overnutrition, called metaflammation, contributes to the common pathogenesis of obesity-related metabolic diseases (2).

Immune cells in metabolic organs, including adipose, liver, and pancreas may sense the changes of micro-environment and interact with local cells, leading to insulin resistance, fibrosis, islet dysfunction, and disrupted metabolic homeostasis (3). The visceral adipose tissues (VAT) contains relatively more immune cells and more abundance of vascular, and direct contact with the liver through the portal circulation. It has been revealed that inflammation in VAT is the predominant source of global metaflammation and thus leads to insulin resistance and T2DM (4). However, the initiation of metaflammation is not entirely settled. Studies on obesity patients and rodent models suggested that excessive nutrients bring lipotoxicity, mitochondrial dysfunction, endoplasmic reticulum stress and hypoxia in adipose tissue, lead to pro-inflammatory cytokine and chemokine secretion, and trigger inflammation infiltration (5).

Anti-inflammatory therapies have the potential to become an effective treatment for obesity-related metabolic diseases (2). Mouse models based on inbred strains are widely used to explore the basic pathophysiological mechanisms; however, due to the tremendous complexity of the inflammation network and the huge gap between human and rodent immunology, the translatability of putative targets is limited (6). Therefore, a more predictive animal model integrating genetic and environmental effects of metabolic disease is urgently needed. Pigs share more physiological, metabolic and inflammatory similarities with humans, this might be the bridge linking experimental treatments and clinical trials (7–9). In metabolic disease research, several transgenic or diet-induced pig models were developed. In 2010, Renner et al. reported a pig model expressing a dominant-negative glucose-dependent insulinotropic polypeptide receptor (*GIPR^dn^*) in the pancreas, with decreased glucose tolerance and reduced β-cell proliferation (10). In 2012, a pig model for permanent neonatal diabetes mellitus was established by the transgene of insulin mutant *INS^C94Y^* (11–13). These transgenic pig models provide valuable insight into drug verification and clinical translation (14).

In this study, three well-recognized T2DM or NAFLD risk genes that contribute to islet dysfunction or liver inflammation were tissue-specific expressed in Bama pigs. GIPR^dn^ and human islet amyloid polypeptide (*hIAPP*) expressed in the pancreas impairs incretin function and increases β-cell apoptosis, respectively (10, 15). Patatin-like phospholipase domain-containing three variant rs738409 C>G p.I148M (*PNPLA3^I148M^*) expressed in the liver was proven to promote liver lipid deposition and increase the recruitment of inflammatory cells in human and mice (16). After 12 weeks of high-fat-high-sucrose diet (HFHSD) intervention, triple-transgenic (TG) pigs exhibited impaired glucose tolerance and more pancreas lipid deposition than wild-type controls (WT). Inflammation infiltration and the accompanied transcription evidence were found in VAT and liver of TG pigs, but not in the WT pigs. Most importantly, this work reports CD8^+^ T cell activation at the preliminary stage of global metaflammation in a pig model, suggesting that TG pig may be an ideal model for the study of human metaflammation related diseases.

## Materials And Methods

### Experimental Design

Bama pigs have been genetically modified by three targeted knock-in T2DM and NAFLD risk genes (*PNPLA3^I148M^-GIPR^dn^-hIAPP*). A total of 10 transgenic males and 10 age- and weight-matched wild-type males were used. Pigs were fed with a control diet until 9 months and a HFHSD (37% sucrose, 53% control diet and 10% pork lard) (17) for the next 12 weeks. After 12 weeks of HFHSD intervention, serum indicators were tested to determine the metabolic status of animals. Bio-banks were constructed of various tissues sampled from TG and WT pigs, including but not limited to pancreas, liver and different types of adipose tissues. Histological analysis was used to observe pathological progression, RNA-seq was next conducted to reveal the molecular signature of tissue metaflammation (**Supplementary Figure 1**).

### Animals

All animal experiments were approved by the Animal Care and Use Committee of the Germplasm Resource Center of Chinese Experimental Minipigs (Institute of Animal Sciences, Chinese Academy of Agricultural Sciences, permit No. IAS2019-12), where the animals were treated humanely following the “Guide for the Care and Use of Laboratory Animals, ISA, CAAS”. Tested pigs were housed in single pens, fed twice daily, provided water *ad libitum*, keeping temperature 16–28°C, relative humidity 40–70%. Surgery to collect pancreatic tissue was implemented under isoflurane anesthesia (3.0% in pure oxygen), ketamine (15 mg/kg i.m.) and xylazine (1 mg/kg i.m.) were used as analgesic during surgery. Other tissue samples were collected after animals were euthanized humanely, and immediately frozen in liquid nitrogen (18).

### Genetic Modification of Bama Pigs

Plasmid vector of CRISPR/Cas9 system targeting pig H11 locus was constructed previously in our laboratory (19). Recombinant HDR donor vector consisted of two expression cassettes was constructed based on pcDNA3.1c (+) (Invitrogen, V790-20), in which human *PNPLA3^I148M^* (NM_025225.3, rs738409, C > G) was driven by porcine liver-specific apolipoprotein E promoter (*ApoEP*), *GIPR^dn^* and *hIAPP* (NM_000415.2) were driven by porcine insulin promoter (*InsP*). cDNA sequences of target genes with additional restriction sites were synthesized (Invitrogen, Thermo Fisher Scientific, Inc.) and assembled into a recombinant vector by restriction enzyme digestion and ligation (19). Porcine fetal fibroblasts (PFFs) of Bama pigs were isolated from day 35 fetuses. CRISPR/Cas9 vector plasmid and HDR donor plasmid were co-transfected into fetal fibroblast. After transfection, cells were re-suspended and cultured 50–100 cells per 10 cm^2^ dish for 10–12 days for no-drug selection. Monoclones were sequenced, *PNPLA3^I148M^-GIPR^dn^-hIAPP* positive clones were selected for somatic cell nuclear transfer and embryo transfer, and reconstructed embryos were transplanted into eight recipient sows with synchronized estrus. More details could be found in our previous study (20). Gene sequences were annotated by a SnapGene software.

### Genotyping of Transgenic Pigs

**Figure 1:**
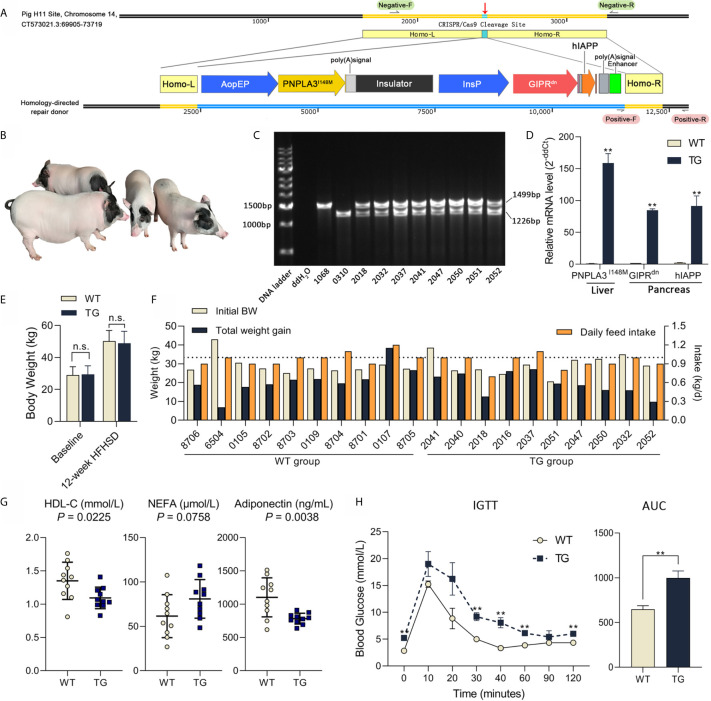
Genetic modification, genotyping and metabolic status of Bama pigs. **(A)** Pig H11 sites on chromosome 14 were chosen for gene knock-in. Liver-specific promoter ApoEP was used for PNPLA3^I148M^ expression and pancreas-specific promoter InsP was used for GIPR^dn^ and hIAPP expression. **(B)** Photo of transgenic Bama pigs. **(C)** PCR analysis of transgenic pigs, 1,499 bp product signed wild-type and 1,226 bp product signed transgene-positive H11 locus. No. 1068 was the wild-type sow, No. 0310 was the transgenic positive founder male, Nos. 2032, 2037, 2041, 2050, 2051 and 2052 were F1 transgenic heterozygotes. **(D)** Relative mRNA expression of PNPLA3^I148M^ in liver and GIPR^dn^ and hIAPP expression in pancreas detected by RT-qPCR. n = 4 per group. **(E)** Average body weight at baseline and after a 12-week HFHSD. n = 10 per group. **(F)** Individual body weight at the start of HFHSD intervention (Initial BW), total weight gain and average daily feed intake during HFHSD intervention of animals. **(G)** Serum biochemical parameters; Symbols represent individual pig. **(H)** Blood glucose during IGTT and calculation of area under curve of IGTT test (0.6 g/kg, ip); n = 4 per group. Data are mean ± SD; statistical difference was performed using Student’s t-test; n.s., not significant, **P < 0.01.

Two PCR primer pairs with different product lengths were designed to distinguish transgenic-positive or transgenic-negative at H11 locus. The forward primer for transgenic-positive product was located at the exogenous enhancer sequence, the corresponding reverse primer was located at the downstream of CRISPR/Cas9 cleavage site on pig genome. The forward and reverse primer for transgenic-negative (wild-type) product were located at the upstream and downstream of CRISPR/Cas9 cleavage site on pig genome, respectively. Genomic DNA extracted from ear tissue was used as a PCR template. Once the target sequence correctly insert H11 locus, a transgenic-positive product of 1,226 bp would be amplified, while the transgenic-negative product of 1,499 bp cannot be amplified because of the large fragment inserted (**Figure 1A** and **Supplementary Table 5**). UV imaging after agarose gel electrophoresis recorded the size of PCR products.

### Intravenous Glucose Tolerance Test

Pigs were fasted for 16 h and injected with 50% glucose at 1.2 ml/kg body weight through ear veins in 2 min. Blood samples were obtained from the ear veins of the contralateral ear. Whole blood glucose was measured at 0, 10, 20, 30, 40, 60, 90 and 120 min using glucose test strips (OneTouch^®^ Ultra^®^, LifeScan Europe).

### Serum Test

Fasted blood glucose was measured with venous blood collected from the auricle (Johnson, Ultra, America). A total of 20 ml blood sample was collected into BD vacutainer tubes (KJ030AS, Kangjian) from the anterior vena cava after overnight fasting. Whole blood was centrifuged at 3,000 rpm for 10 min at 4°C to isolate serum. Serum concentrations of alanine aminotransferase (ALT), aspartate aminotransferase (AST), total cholesterol (TC), triglyceride (TG), low density lipoprotein cholesterol (LDL) and high density lipoprotein cholesterol (HDL) were measured with an AU480 auto-analyzer (Olympus Co.). Non-esterified fatty acid (NEFA) was measured by an enzymatic assay kit (A042-2-1, NJJCBIO). Insulin (10-1200-01, Mercodia), glucagon (10-1281-01, Mercodia), CRP (DY2648, R&D), leptin (DLP00, R&D) and adiponectin (DRP300, R&D) level were determined using enzyme linked immunosorbent assay.

### Histopathological Examination

Eight TG pigs and five WT pigs were sampled. Tissue samples were collected from the pancreas, liver, SAT (backfat) and VAT (greater omentum) and fixed in 4% paraformaldehyde. Fixed tissue was embedded with paraffin and sliced into continuous sections. Hematoxylin–eosin (HE) staining was used to evaluate tissue morphology. Immunohistochemical staining for insulin and glucagon were performed using insulin antibody (ab181547, Abcam) and glucagon antibody (ab36232, Abcam). Histological sections were panoramically scanned and read by Motic DSAssistant Lite software. ImageJ software was used for statistics of islet size, pancreas lipid deposition, adipocyte size and hepatic inflammation, in systematic random selected views. The size of islets were measured in insulin stained sections (21).

### RNA Sequencing

Total RNA was extracted using TRIzol Reagent following the manufacturer’s protocol (Invitrogen) from liver and VAT of eight TG pigs and five WT pigs. RNA samples with good quality (RIN >6.80) were used for cDNA library construction. RNA sequencing was implemented using Illumina HiSeq X Ten (Novogene Bioinformatics Technology Co., Ltd., Beijing, China). Clean reads were aligned to *Sus scrofa* genome version Sscrofa11.1. The number of hits of each gene were quantified by HTSeq software, the transcripts abundance was calculated by fragment per kilobase of transcript per million fragments mapped (FPKM) using DESeq2. Differently expressed genes were identified by the threshold *P <*0.05 and |log2 fold-change|>1. Kyoto Encyclopedia of Genes and Genomes (KEGG) pathway enrichment analysis and immunologic signatures enrichment were conducted by gene set enrichment analysis (GSEA), gene set databases were obtained from the MSigDB database (c2.cp.kegg.v7.2 for KEGG enrichment, c7.all.v7.2 for immunological signatures).

### RT-qPCR

Total RNA was extracted from the pancreas, liver and visceral fat of five pigs each group using TRIzol Reagent (Invitrogen). cDNA template for RT-qPCR was synthesized using a RevertAid First Strand cDNA Synthesis Kit (K1662, Thermo Fisher Scientific). RT-qPCR was performed on a QuantStudioTM3 Real-time PCR Instrument (Thermo Fisher Scientific) with PowerUpTM SYBRTM Green Master Mix (A25742, Thermo Fisher Scientific) following the manufacturer’s instruction. Pig 18s ribosome was used as a reference gene. Primers used in RT-qPCR were designed by Primer3 (**Supplementary Table 5**). Relative mRNA expression was calculated by the delta delta CT method.

### Protein Sequence Alignment

Protein sequences were downloaded from the UniProt database (https://www.uniprot.org/). Sequences were aligned by ClustalX 2.1 and paint by Genedoc.

### Statistical Analysis

Prism 8 and R studio based on R version 3.6.3 were used for statistical analysis and plotting. Two-tailed Student’s t-tests were used for comparison between groups. The Benjamini–Hochberg procedure was used to calculate false discover rate. Data were expressed as mean ± standard deviation (SD), *P*-value <0.05 was considered significant. Protein–protein interaction analysis were conducted with the String website (https://string-db.org/), and visualized by Cytoscape v3.6.1 software.

## Results

### Genetic Modification and Genotyping of Bama Pigs

This work aims at constructing an ideal large animal model for metabolic diseases. To target the expression of *PNPLA3^I148M^* in the liver and *GIPR^dn^* and *hIAPP* in the pancreas, a homologous oriented repair donor was established with *PNPLA3^I148M^* under the control of liver-specific *ApoE* promoter, followed by an insulator, and *GIPR^dn^* and *hIAPP* under the control of pancreas-specific insulin promoter (**Figure 1A**). After nuclear transfer embryo transplantation, one male founder animal (No. 0310) was born. This founder pig was mated to six wild-type sows for breeding, and 51 F1 offspring (25 female and 26 male) were obtained. PCR analysis showed that the founder pig was homozygous mutant with a single 1,226 bp band at the H11 site and all F1 offspring were transgenic heterozygote, with both transgenic (1,226 bp) and wild type (1,499 bp) alleles, according to Mendelian rules (**Figures 1B, C** **)**. Real-time quantitative PCR (RT-qPCR) analysis showed that *PNPLA3^I148M^* was highly expressed in the liver; *GIPR^dn^* and *hIAPP* were highly expressed in the pancreas of TG pigs, while these target genes were almost absent in WT pigs (**Figure 1D**). During 12 weeks of HFHSD intervention, average body weights were not different between the two groups (**Figure 1E**, *P >*0.05). Individual data including initial body weight, total weight gain and daily feed intake are shown in **Figure 1F**.

### Serum Biochemical Parameters and Glucose Intolerance in TG Pigs

To evaluate the disruptive effect of target genes on pig metabolic homeostasis, serum indicators related to glucose metabolism, lipid metabolism, cholesterol metabolism, liver injury, adipokine secretion and infection were tested after 12 weeks of HFHSD (**Supplementary Table 1**). No significant difference were observed in insulin, glucagon or hepatic aminotransferase. However, serum concentration of high density lipoprotein cholesterol (HDL-C; *P* = 0.0225) was significant lower in TG vs. WT samples, while the concentration of non-esterified fatty acid (NEFA; *P* = 0.0758) seemed higher in TG pigs (132% of WT) (**Figure 1G**). Adiponectin concentration in circulation was significant lower in TG pigs (71% of WT; *P* = 0.0038) (**Figure 1G**). C-reactive protein (CRP; *P*=0.5546) did not show significant difference between groups, suggesting that there was no acute inflammation.

TG pigs exhibited impaired glucose tolerance after diet intervention, with significantly higher blood glucose levels at 0, 30, 40, 60 and 120 min (*P <*0.05) and delayed recovery period (*P <*0.05) during intravenous glucose tolerance test (**Figure 1H**). The area under the glucose curve of the TG pigs in the glucose tolerance test was 54% larger (*P <*0.01) than that of the WT pigs (**Figure 1H**). Therefore, compared with WT, TG pigs had altered lipid metabolism and glucose intolerance after HFHSD, thus are more sensitive to energy overload.

### Accumulated Lipid Infiltration and Changed Islet Feature in the Pancreas

**Figure 2:**
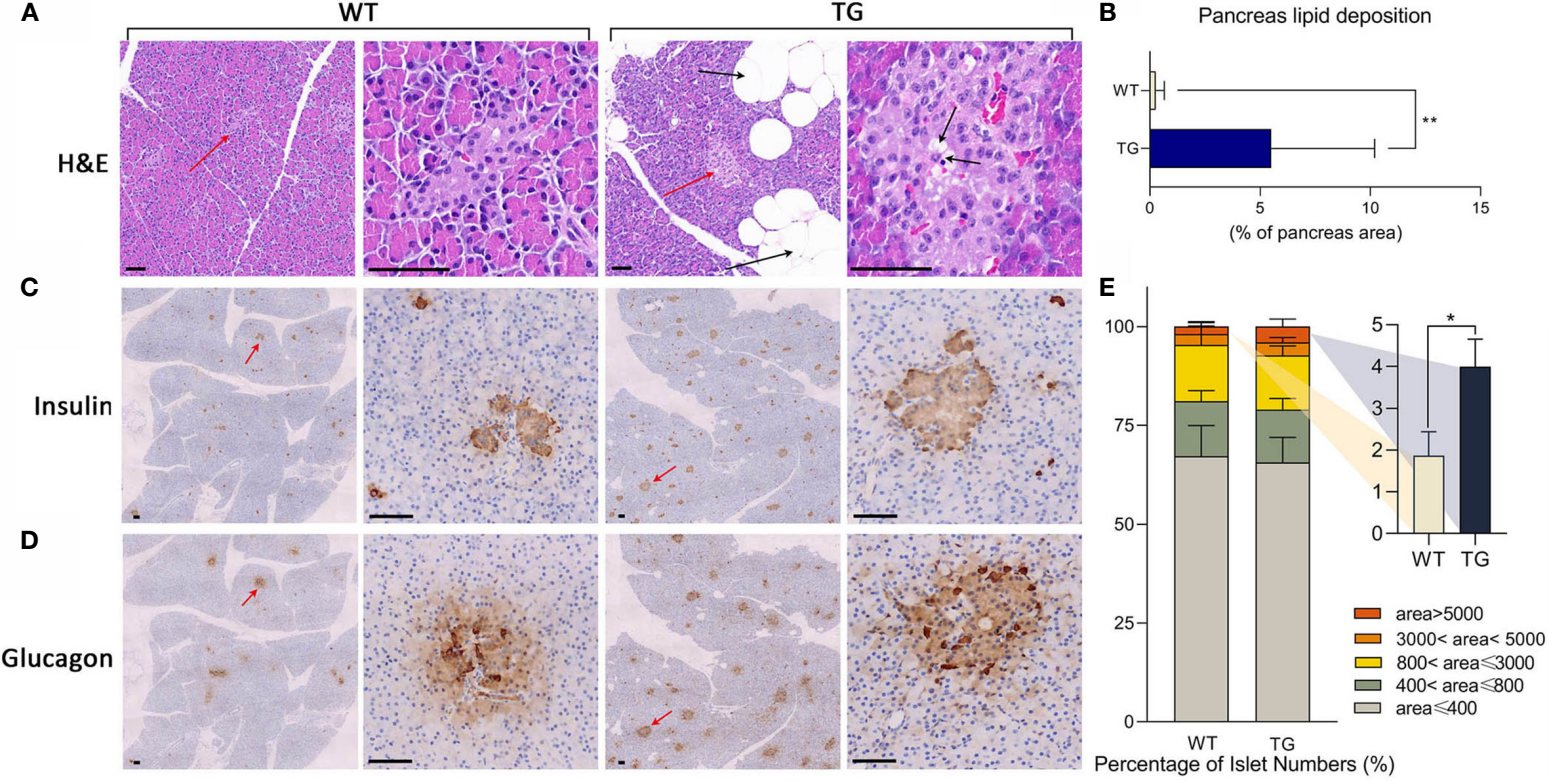
Histological analysis of pancreas. **(A)** Representative images of pancreas HE staining. Islets were pointed out by red arrows and presented in the second and the fourth column. Lipid drops were pointed out by black arrows. Scale bars, 50 μm. **(B)** Statistics of the percentage of lipid deposition in the pancreas. **(C, D)** Immunohistochemical staining of insulin **(C)** and glucagon **(D)** in two continuous sections. Islets were pointed out by red arrows and presented in the second and the fourth column. Scale bars, 50 μm. **(E)** Statistics of islet area by insulin staining. *P < 0.05, **P < 0.01.

To investigate the histopathological changes in the pancreas, hematoxylin–eosin (H&E) staining was performed. A significant adipose deposition was found in the pancreas of the TG pigs, while almost no obvious lipid infiltration could be observed in the WT pigs (*P <*0.01) (**Figures 2A, B** and **Supplementary Figure 2**). Compared with the clean pancreas and islets in WT pigs, we found large adipocytes infiltration among pancreatic lobules, lipid deposition around acinar cells and lipid drops inside islets in TG pigs (**Figure 2A**). The morphology and hormone secretion of the same islet was exhibited by insulin and glucagon staining of continuous sections (**Figures 2C, D** and **Supplementary Figure 3**). We measured islet size on insulin stained pancreatic sections (21). To avoid statistical bias caused by calculating only cross-sections, average 10 randomly selected views of each pancreatic section were analyzed. The proportion of large islets (area >5,000 pixels in 10× section) in TG pigs was twice than that of WT pigs (*P <*0.05) (**Figure 2E**). Meanwhile, TG pigs had extremely intense staining of glucagon and insulin, indicating a high concentration of the two hormones in the islet microenvironment. In contrast, WT pigs did not show excessive glucagon secretion. These results indicated that the pancreatic function of TG pigs was changed.

### Inflammatory Infiltration in VAT and Liver

**Figure 3:**
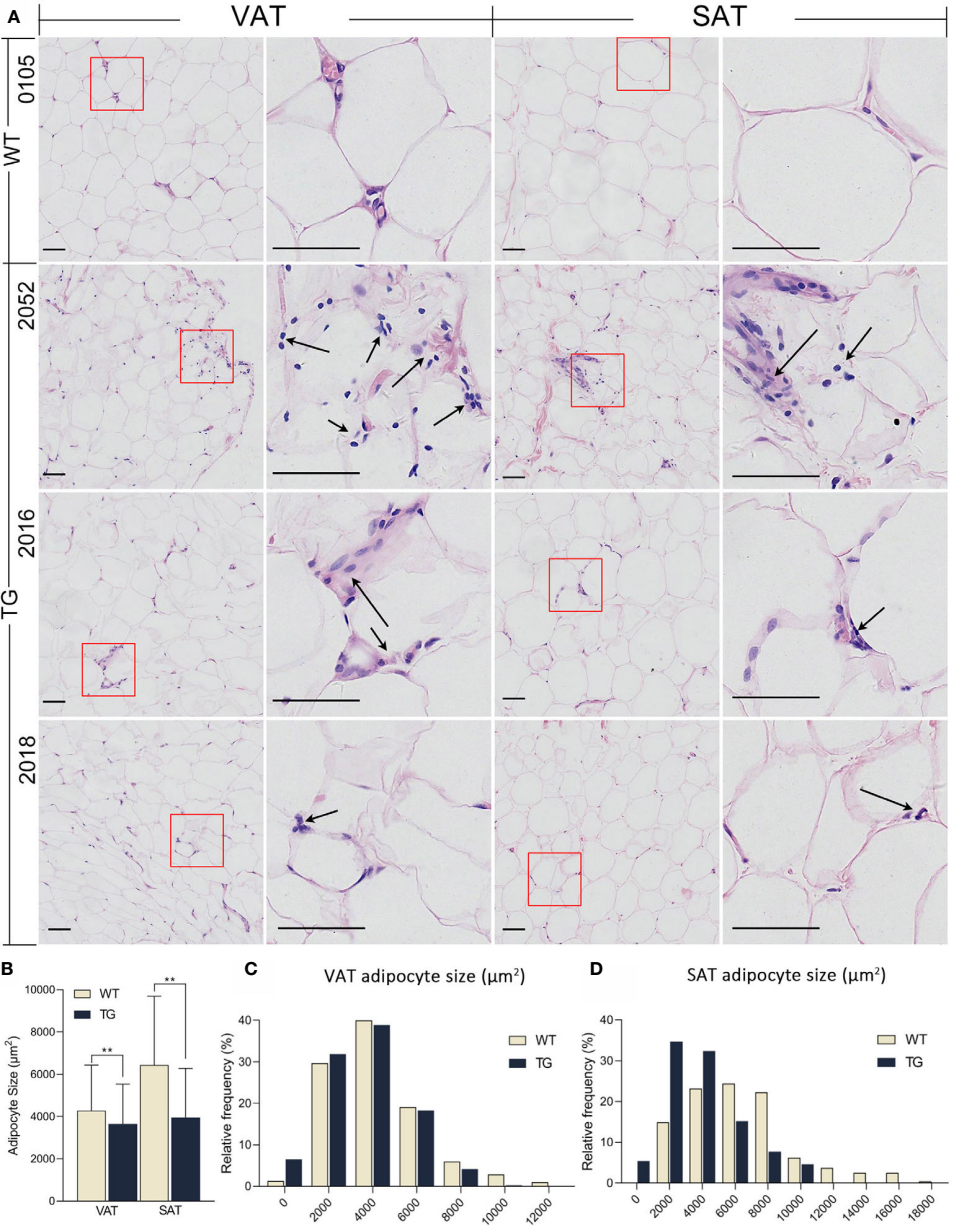
HE staining of adipose tissues. **(A)** Visceral adipose tissue (VAT) collected from greater omentum and subcutaneous adipose tissue (SAT) collected from backfat were used for HE staining. Immune cells were pointed out by black arrows. Scale bars are 50 μm. **(B)** Statistics of adipocyte size. Three randomly selected views of each sample were used for statistics, n = 5 per group. **(C, D)** Frequency distribution of adipocyte size in VAT **(C)** and SAT **(D)** from the two groups. **P < 0.01.

The liver and adipose tissues are important metabolic tissues and the frequent place of metaflammation, so we speculate that metabolic changes would lead to metaflammation in the liver and adipose tissues from TG pigs. Thus, H&E staining was also performed on VAT, subcutaneous adipose tissue (SAT) and the liver to investigate their histopathological changes (**Figure 3**). TG pigs had accumulated inflammatory cells that have dark nuclear staining and a few cytoplasmic staining in VAT and SAT, while few inflammatory cells were found in adipose tissue from WT pigs (**Figure 3A**). Comparing VAT and SAT of TG pigs, the former had more inflammatory cells gathered around adipocytes. Adipocyte size in VAT and SAT were suggested by the cross-section area of adipocytes in randomly selected view of H&E stained sections. The average size of adipocyte in VAT and SAT from TG pigs were significantly smaller (*P <*0.01) (**Figure 3B**), and both fats displayed more heterogeneity than those from WT pigs (**Figures 3C, D** **)**.

**Figure 4:**
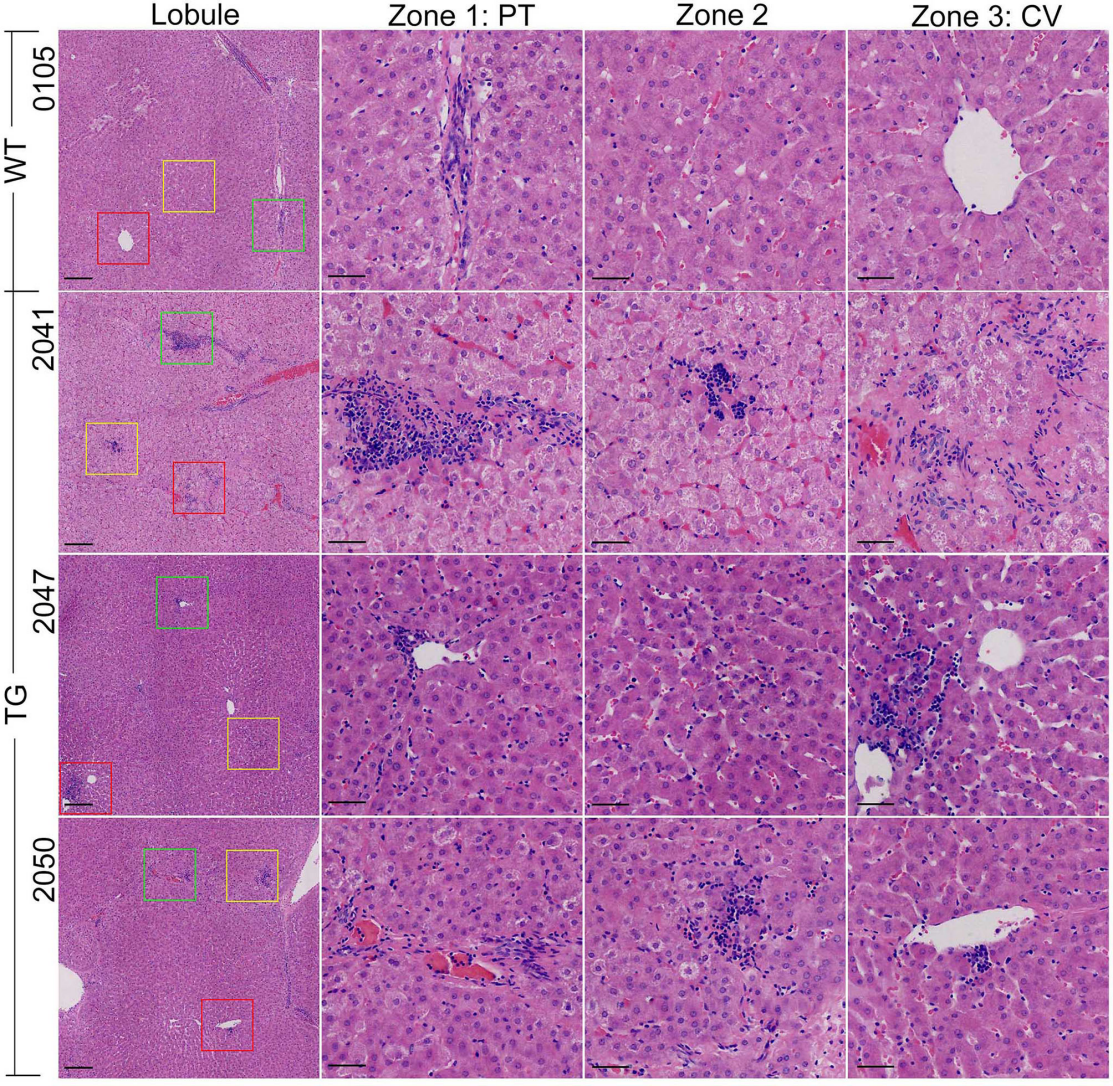
HE staining of the liver. The vision selected from lobule view were marked with different colored boxes: green, zone 1; yellow, zone 2; red, zone 3; PT, portal area; CV, central vein. Scale bars are 100 μm in the lobule views and 50 μm in zones 1–3 views.

Incidentally, obvious hepatic inflammation was found in TG pigs, but not in WT pigs (**Figure 4**). Statistical calculation of inflammation cells per 20× magnified view showed 50% significantly higher inflammatory cells in the liver from the TG pigs (*P* = 0.0182). Infiltrated inflammatory cells accumulated and formed inflammatory foci in the liver of TG pigs, while few resident macrophages (Kupffer cells, KCs) were regularly distributed in the perisinusoidal space of WT pigs. For each 20× magnified view, two or more inflammatory foci could be found at different zones in the liver sections from of TG pig, including portal area, lobules, and central vein (**Figure 4**). Connecting with the unchanged serum CRP level (**Supplementary Table 1**), these results suggested that chronic inflammation occurred in adipose tissues and the liver of TG pigs.

### Transcriptome Profiles of Metaflammation in VAT and Liver of TG Pigs

To generate an unbiased view of the inflammatory process and immune-metabolic cross-talk in VAT and liver in pigs after HFHSD intervention, RNA sequencing (RNA-seq) analysis was conducted (n = 8 for TG group, n = 5 for WT group). The average depth of RNA sequencing was 62.8 million reads per sample. Transcripts of 24,355 and 23,091 genes were detected and quantified in VAT and liver, respectively. By the threshold *P <*0.05 and |log2FC|>1, 428 and 364 genes were significantly up- or down-regulated in VAT; while 409 and 217 genes were significantly up- or down-regulated in the liver, in the TG pigs compared to the WT pigs (**Supplementary Figures 4A, B** **)**.

**Figure 5:**
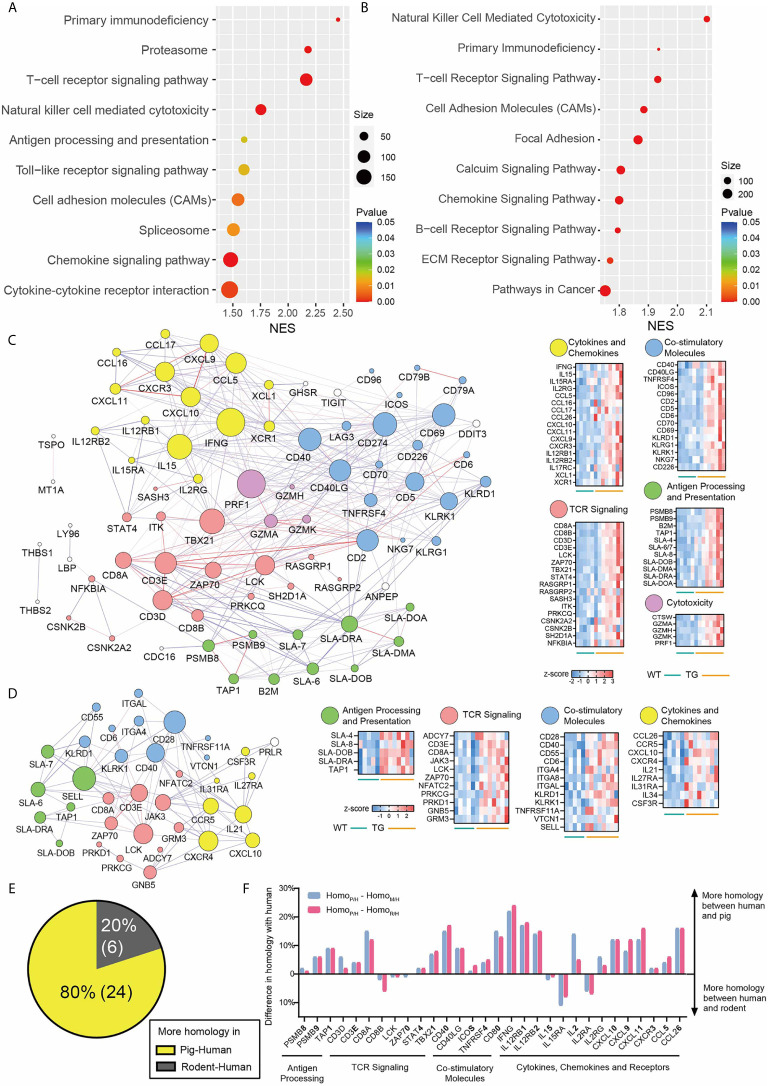
Transcriptome profiles of VAT and liver. **(A, B)** GSEA enrichment of significant up-regulated KEGG pathways in VAT **(A)** and liver **(B)** of TG pigs. **(C, D)** Protein–protein interaction analyze and heatmap of core enrichment genes in VAT **(C)** and liver **(D)** of TG pigs. Genes were clustered by their function in CD8^+^T cell activation: antigen processing and presentation (green), TCR signaling (red), co-stimulation and co-inhibition (blue), cytokine and chemokine signals (yellow) and cytotoxicity (violet) heatmap of core enrichment genes in VAT. **(E, F)** 30 CD8^+^ T cell-related genes that differentially expressed in TG vs. WT pigs were used for protein sequence alignment. **(E)** Percentage of genes that have more homology between human and pig (yellow) and genes that showed more homology between human and rodent (gray). **(F)** The difference in homology with human was calculated by homology between pig and human (Homo_P/H_) minus homology between mouse and human (Homo _M/H_), shown in pink column; and calculated by homology between pig and human (Homo _P/H_) minus homology between rat and human (Homo_R/H_), shown in blue column.

To assess the underlying functional pathways, gene set enrichment analysis (GSEA) was conducted using the Kyoto Encyclopedia of Genes and Genomes (KEGG) gene set database. Significant up-regulated pathways were identified by *P <*0.05 and Benjamini–Hochberg adjusted false discover rate <25%, ranked by normalized enrichment score (**Figures 5A, B** and **Supplementary Table 2**). The enrichment of inflammatory pathways indicated VAT inflammation of TG pigs, including primary immunodeficiency, proteasome, T-cell receptor (TCR) signaling pathway, natural killer (NK) cell-mediated cytotoxicity, antigen processing and presentation, Toll-like receptor signaling pathway, cell adhesion molecules, chemokine signaling pathway, and cytokine-cytokine receptor interaction. Pathways related to retinol metabolism and fatty acid metabolism were also significantly enriched in VAT from TG pigs (**Figure 5A**). Likewise, inflammation related pathways were also enriched in liver from TG pigs, including NK cell mediated cytotoxicity, primary immunodeficiency, T-cell receptor signaling pathway, cell adhesion molecules and chemokine signaling pathway (**Figure 5B**). These data suggested that genes up-regulated in VAT and liver from TG pigs are closely associated with inflammation.

To specifically focus on immune processes and cell types, we conducted GSEA on VAT with immunologic gene sets database ImmuneSigDB (c7.all.v7.1) that are defined from 389 immunology studies (22). ImmuneSigDB provided us transcriptome signatures of diverse cell states in immunology. Among the total of 4,872 immunologic gene sets, 614 were significantly enriched in the TG pigs, while only 13 gene sets were enriched in WT pigs (**Supplementary Table 3**). The enrichment in gene set GSE9650 (genes down-regulated in naïve CD8^+^ T cells versus effector CD8^+^ T cells) and GOLDRATH (genes down-regulated in naïve CD8^+^T cells versus memory CD8^+^T cells) suggested that comparing naïve CD8^+^ T cells and memory/effector CD8^+^ T cells, transcriptome signatures in VAT of TG pigs are more consistent with the two groups. Therefore, memory and/or effector CD8^+^ T cells might constitute the majority CD8^+^ T cell in VAT from TG pigs. Likewise, the enrichment in gene set GSE3039 (genes down-regulated in NKT cells versus CD8A T cells) indicated that comparing NKT cells and CD8αα T cells of the innate immune system, the latter might be more abundant in VAT of TG pigs (**Supplementary Figure 4C**). The enrichment of gene sets related to CD8^+^ T cells in TG pigs indicated that CD8^+^ T cell is involved in VAT metaflammation of TG pigs.

### Molecular Features of CD8^+^ T Cell Activation in VAT and Liver

It has been well established that the adaptive immune system is critically involved in obese fat inflammation. CD8^+^ T cells are essential for adipose inflammation. They are activated in obese adipose tissue, then in turn recruit and activate macrophages (4). A recent study in obese mice confirmed that class I major histocompatibility complex (MHC-I) associated peptide presentation of adipocytes toward CD8^+^ T cell was reshaped, which may trigger adipose metaflammation (23). Functional enrichment gave us an overall description of metaflammatory happenings in VAT and liver of TG pigs, suggesting that CD8^+^ T cell was a central player in early-stage metaflammation. To better understand the process of CD8^+^ T cell activation, protein-protein interaction analysis was conducted using core enrichment genes in significant immunological pathways. Genes were clustered by their direct function on CD8^+^ T cell activation. The biological processes involved in CD8+ T cell activation were similar in the liver and VAT from TG pigs (**Figures 5C, D** **)**. The expression of representative genes was further detected by RT-qPCR (**Supplementary Figures 4C, D** **)**.

TG pigs had enhanced endogenous immunopeptide processing and peptide presentation through MHC-I, suggested by the up-regulation of immunoproteasome typical 20S subunit beta 8/9 (*PSMB8/PSMB9*), antigen peptide transporter 1 (*TAP1*), and swine leukocyte antigen (*SLA*) that encode MHC-I molecules (**Figure 5C**, green bubbles) (24). MHC-I molecules that up-regulated in TG pigs included *SLA-4* that belongs to classical MCH-1a, and *SLA-6*, *-7* and *-8* that belong to non-classical MHC-Ib. As a strong sequence homology between class Ia SLA genes and human leukocyte antigen (HLA) counterparts, the three SLA class Ib genes (*SLA-6*, *-7* and *-8*) with high transcription level in TG pigs were also believed to play similar roles with non-classical HLA genes (*HLA-E*, *-F* and *-G*) (25). Notably, *SLA-4*, which is traditionally considered as a pseudogene, was detected at a dramatically high mRNA level in TG pigs (average FPKM 58.87, eight times than in WT pigs).

After antigen recognition *via* MHC-I molecules, activation of TCR signaling (**Figure 5C**, pink bubbles) was indicated by up-regulation of TCR-CD3 complex with their co-receptors (*CD3D*, *CD3E*, *CD8A* and *CD8B*). The predominant down-stream immune signaling kinases, including leukocyte c-terminal Src kinase (*LCK*) and zeta chain of TCR associated protein kinase 70 (*ZAP70*) were increased. Then signal transducer and activator of transcription 4 (*STAT4*) and T-box transcription factor 21 (*TBX21*, also refers to *T-bet*), the crucial transcriptome factors of pro-inflammatory T cell activation and cytokine release, were also up-regulated in the TG pigs.

Co-stimulatory molecules on the cell surface of both APCs and naïve T cells could mediate crosstalk between T cells and immune or non-immune cells, direct T cell as well as other immune cell responses and cytokine production (26). Core enrichment genes in cell adhesion molecules pathway introduced a series of co-stimulatory molecules that contribute to T cell activation (**Figure 5C**, blue bubbles), including killer cell lectin like receptor K1 (*KLRK1*), inducible T cell co-stimulator (*ICOS*), T-cell-activated increased late expression protein (*CD96*), early T-cell activation antigen P60 (*CD69*), tumor necrosis factor receptor superfamily member 4 (*TNFRSF4*, *OX40*), TNFRSF5 (*CD40*), and CD40 ligand (*CD40LG*). These results suggested that in VAT of TG pigs, cell-interaction through co-stimulatory molecules is another important process that mediates T cell activation, especially for effector CD8^+^ T cells. Interestingly, programmed cell death protein 1 (*PDCD1*), its ligand (*CD274*), lymphocyte activating 3 (*LAG3*), and the co-inhibitory molecules of antigen-activated T-cells, were increased contemporary, which generally lead to T cells (especially CD8^+^ T cells) exhaustion and T cell tolerance (27). We guess that this may occur due to the chronic exposure of T cells to antigens, which was similar to T cell exhaustion in tumor microenvironments.

As the consequence of activating the pro-inflammatory TCR signaling pathway, the expression of CD8^+^ T cell-related cytokines and chemokines were elevated in VAT of TG pigs (**Figure 5C**, yellow bubbles). The high-lighted one was type II interferon (*IFNG*), the key mediator between the innate and adaptive immune responses. IFNγ is produced by activated T cells and NKs, and promotes macrophage polarization, pro-inflammatory cytokine secretion, and antigen presentation during CD8^+^ T cell priming (4). C-X-C motif chemokine receptor 3 (*CXCR3*) and its ligand *CXCL9*, *CXCL10*, and *CXCL11* were accordingly increased, as the latter three chemokines were regarded as IFNγ-inducible monokine, IFNγ-inducible 10 kDa protein and IFN-inducible T cell a-chemoattractant, respectively. X–C motif chemokine ligand 1 (*XCL1*) and its receptor *XCR1* are specifically chemotactic for T cells, XCL1 is secreted by activated CD8^+^ T cells and is essential for their maximal priming and expansion (4). C–C motif chemokine ligand 5 (*CCL5*) is one of the major chemokines produced by CD8^+^ T cells, while *CCL16* is the chemotactic factor for lymphocytes and monocytes. These results indicated pro-inflammatory crosstalk between immune cells in VAT of TG pigs. T lymphocytes, NKs, monocytes and other immune cells are activated, then secrete cytokines and chemokines, and in-turn enhance inflammatory signals.

Another trail to CD8^+^ T cell activation in VAT of TG pigs was the elevation of cytotoxic molecules (**Figure 5C**, violet bubbles), including granzymes (*GZMA*, *GZMH* and *GZMK*), perforin (*PRF1*), and cathepsin W (*CTSW*). Although these genes were enriched in NK cell-mediated cytotoxicity KEGG pathway, the expression of a specific surface marker of NKs (for example, *CD56*, *CD27*, or *CD11b*) was not up-regulated in VAT of TG pigs (*P >*0.05). Connecting with the above molecular evidence of CD8^+^ T cell activation, we suggest that the cytotoxic effect in VAT of TG pigs might occur mainly due to effector CD8^+^ T cells.

Similar to the transcriptome result of VAT, the metaflammation responses in the liver of the TG pigs included antigen processing and presentation, TCR signaling, co-stimulatory molecules, pro-inflammatory cytokine and chemokine signals (**Figure 5D**). These results are in line with the fact that significant inflammation foci were generated only in TG pigs, and provided molecular evidence for initial inflammation that occurred at this stage. In summary, CD8+ T cells activation is the main feature of early stage metaflammation in VAT and liver from TG pigs.

### Other Immune Cells Activation in VAT and Liver

Immune response has a complicated regulatory network, where many molecules were shared by activated pathways of different immune cells. For example, *KLRK1* and its family members *KLRD1*, *KLRG1* are crucial molecules for cell recognition by NKs and CD8^+^ T cells. They are expressed on both NKs and effector CD8^+^ T cells, regulating cytotoxic function. Although we suggested CD8^+^ T cell as the driver of cytotoxicity in VAT, we could not deny the role of NKs. The surface marker of B cells, including *CD79A* and *CD79B* pointed that B cells might also increase in VAT (**Figure 5C**, blue bubbles).

Class II MHC molecules including *SLA-DRA*, *-DOA*, *-DOB*, and *-DMA* were also up-regulated in TG pigs (**Figure 4C**, green bubbles). MHC-II molecules are generally expressed on professional APCs, including macrophages, dendritic cells and B cells (26). It has been reported that adipocytes can also act as non-professional APCs in diet induced obese (DIO) mice through MHC-II and co-stimulatory molecules, which induces CD4^+^ Ths differentiation toward pro-inflammatory phenotype (28). In line with studies on human and mice, in HFHSD TG pigs, *SLA-DRA* showed a considerable high expression in VAT (average FPKM 106.62, 1.6 times than WT pigs) compared with other *SLA* members, indicating that *SLA-DRA* may not restrict on professional APCs, but also exist on VAT adipocytes. Meanwhile, Ths were activated through interaction between MHC-II antigen and CD4^+^ TCR complex. As CD4^+^ Ths are a major source of pro-inflammatory cytokines and chemokines, we suggest that CD4^+^ Ths were also increased in VAT of TG pigs.

Similar to the transcriptome result of adipose tissue, a series of pro-inflammatory cytokines, namely, *IL34*, *CCL26* and cytokine receptors, namely, *CCR5, CSF3R* and *IL31RA*, which were proven to help recruit monocytes and macrophages, had higher expression in the liver from TG pigs (**Figure 5D**, yellow bubbles). Genes involved in MHC-II (*SLA-DOB* and *SLA-DRA*) mediated antigen presentation were also increased (**Figure 5D**, green bubbles), suggesting activated interaction between liver APCs and CD4^+^ T cells in the liver from TG pigs.

### Metabolic Stress in VAT and Liver of TG Pigs

In order to identify whether metaflammation is related to changes in metabolism-related genes, we next focused on the most significantly expressed genes between the two groups. Significantly up-regulated genes in VAT from TG pigs included a series of genes that are associated with T2DM and related complications (**Supplementary Figure 4A**); for instance aquaglyceroporin 3 (*AQP3*), retinol binding protein 7 (*RBP7*), angiopoietin-like 4 (*ANGPTL4*), and metallothionein 2a (*MT2A*). Genes involved in the regulation of metabolic state and inflammation were significantly changed in VAT from TG pigs. For example liver receptor homolog 1 (LRH-1, encoded by *NR5A2*) and immunoglobulin superfamily containing leucine-rich repeat (*ISLR*) were significantly down-regulated in TG pigs. The secretion of adipokines in VAT from TG pigs had changed: the small membrane proteolipid neuronatin (*NNAT*) that involved in the development, glucose metabolism, inflammation and C-type lectin domain-containing membrane glycoprotein layilin (*LAYN*) that promotes CD8^+^ T cells activation were significant up-regulated, while apelin (*APLN*) that facilitate glucose metabolism were down-regulated in VAT of TG pigs (**Supplementary Figure 4A**). These results suggested that metabolic status and adipokine secretory function of VAT were disturbed in TG pigs.

Metabolic pathways, including oxidative phosphorylation, glutathione metabolism, drug metabolism through cytochrome P450, and citrate cycle TCA cycle, were significantly down-regulated in the liver of the TG pigs (**Supplementary Table 2**). Significantly down-regulated genes included crucial enzymes that are catalyzing liver glutathione metabolism GSH S-transferase (*GSTA1* and *GSTA4*) and GSH peroxidase (*GPX3*), members of P450 enzyme families *CYP1A2* and *CYP2A19*, and mitochondrial function-related gene vacuolar protein sorting 13 homolog D (*VPS13D*) and *CDGSH* iron sulfur domain 3 (*CISD3*) (**Supplementary Figure 4B**). These results are highly consistent with the changes in the liver of NAFLD and T2DM patients, including reduced TCA cycle, increased oxidative stress, cytochrome P450 disordered and mitochondrial dysfunction (29), suggesting that TG pigs were less likely to maintain metabolic stability under HFHSD induction.

### Enrichment of Pathways in Cancer in the Liver

NASH is related to the increased risk of hepatocellular carcinoma (HCC). Through GSEA on liver, we found that HCC-related genes were correspondingly changed in the early stage of liver metaflammation. Cancer pathways were enriched in liver of the TG pigs, including glioma, chronic myeloid leukemia, melanoma, and prostate cancer (**Supplementary Figure 4B** and **Supplementary Table 2**). The extracellular matrix (ECM) receptor signaling pathway was also up-regulated, the expression of laminin subunit 3 (*LAMA3*), integrin subunits (*ITGA8* and *ITGA4*), and collagen subunits (*COL21A1*) were increased, suggesting the changes in cell adhesion and migration. The expression of genes that participate in HCC development changed accordingly. Typical ones as β-defensin-1 (*DEFB1*), growth arrest and DNA damage-inducible gamma (*GADD45G*), HSP70 binding protein 21 (encoded by *TTC36*) that protect the organism from and down-regulated in HCC or multiple tumor types, were significantly decreased in liver of TG vs. WT pigs. In contrast, oncogenic genes in HCC, including brain-expressed X-linked 4 (*BEX4*), Golgi membrane protein 1 (*GOLM1*), DNA/HSP40 homolog superfamily C member 6 (*DNAJC6*), were significantly up-regulated in TG vs. WT pigs. Additionally, genes that involved in hepatic stellate cell activation and fibrosis were increased in TG pigs, including the deubiquitinase ubiquitin C-terminal hydrolase 1 (*UCHL1*) and integrin α8 (*ITGA8*) (**Supplementary Figure 4B**). These results suggested that after metaflammation happenings that lead our attention to NASH in the liver, the risk of HCC might also increase in the TG pigs (30).

### High Protein Alignment of Inflammatory Genes Between Porcine and Human

A consensus view is that after non-human primates, porcine shared most structural and functional similarity with the human immune system (31). Thirty inflammatory genes that involved in CD8+ T cell function were identified at the transcript level in metaflammation tissues of TG pigs, including VAT and liver. These genes were highly corresponding to genes highlighted in human metabolic diseases. Therefore, we further compared protein sequences of these genes in human, pig, mouse, and rat (**Supplementary Table 4**). Approximately 24 of 30 analyzed genes had higher protein sequence similarity between human and pig, while only four or two genes were more similar between human and mouse or rat, respectively (**Figure 5E**). The average protein conservation of these genes in human and pig was 13% higher than that in human and mice. For example, CD40, one important co-stimulatory molecular that up-regulated in both VAT and liver of TG pigs, has 73% protein sequence alignment between human and pig, while it shows 58 or 56% sequence alignment between human and mouse or rat (**Figure 5F**). These results further suggest that TG pigs have unique advantages in precision medicine and targeted drug development.

## Discussion

### Triple-Transgenic Pigs as Promising Model for Metaflammation Related Disease

Metaflammation is a common issue in the progression of obesity-related diseases; however, the initiating mechanism is complicated and has not been fully understood. As an ideal experimental animal for metabolic diseases, pig models of metaflammation will help to explore molecular markers and develop therapeutic targets in the research of such diseases. The main difficulty of developing pig models is their high tolerance of overnutrition (32). In our previous study, pigs suffered from 23 months’ HFHSD showed only mild metabolic imbalance or organ injury (17, 33, 34). In this study, TG pigs that express well-proven risk genes of T2DM and NAFLD displayed insulin resistance by the influence of genetic modification.

The dysfunction form of *GIPR* (*GIPR^dn^*) and human *IAPP* gene (*hIAPP*) were expressed in pancreas of TG pigs. Genetic variation of *GIPR* in human was considered to associate with impaired oral glucose tolerance and insulin responses (35). Renner et al. generated a transgenic pig model expressing *GIPR^dn^* in pancreas islets *via* lentiviral transgenesis. The *GIPR^dn^* pigs had impaired insulin secretion in response to GIP, and exhibited impaired glucose tolerance, reduced β-cell proliferation and reduced β-cell mass (10). Triple-transgenic pigs had impaired glucose tolerance, which was consistent with our expectation. However, there was no significant reduction in β-cell mass in triple-transgenic pigs. Fasted blood glucose level and insulin level did not show significant difference at this stage. This could be intelligible, as no β-cell toxic effect nor islet neogenesis disruption in young *GIPR^dn^* transgenic pigs, and their β-cell mass were reduced in an age-related manner (10). In triple-transgenic pigs under short-term dietary intervention, the larger islet size might due to compensatory islet proliferation at young age (36).

Amyloid aggregates in human β-cell are a causative factor of T2DM, while neither porcine nor rodent *IAPP* was prone to form amyloid aggregates (37). According to the results on rodent models, expression of *hIAPP* leads to β-cell apoptosis and the development of islet amyloid with a threshold-dependent manner (38). Heterozygous *hIAPP* transgenic rat (HIP rat) developed diabetes after 6–12 month old, while homozygous HIP rat spontaneously developed diabetes in the first two month of life (39). Pigs have a longer life cycle than rats, so in the current stage, although *hIAPP* expression was detected, we did not find islet amyloid deposition in triple-transgenic pigs. Further evaluation of the long-term effect of *hIAPP* and *GIPR^dn^* on islet of triple-transgenic pigs is still needed.

The *PNPLA3^I148M^* that expressed in TG pigs liver is an independent risk factor for liver diseases (16). A study on people carrying the *PNPLA3^I148M^* variant suggested that this variant is strongly associated with hepatic steatosis and inflammation (16). In a study on NAFLD children, all patients with the rs738409 genotype (homozygote at two alleles) have severe steatosis, inflammation and NASH, while sample steatosis mainly occurred in patients who did not carry the *PNPLA3^I148M^* variant (40). In vivo and *in vitro* models carrying PNPLA3^I148M^ indicated that expression of this variant leads to hepatic steatosis, changes hepatic lipid composition and modulates the fibrogenic phenotype of hepatic stellate cells (HSCs), further contributes to pro-inflammatory condition in the liver (16, 41, 42). In our triple-transgenic pig model, metaflammation in the liver was notable, which will be discussed in detail below.

Following 12 weeks of HFHSD, both groups of pigs had the same degree of weight gain. Although 12 weeks of HFHSD intervention did not cause immediate organ injury, the metabolic condition has already shifted, providing an initial fuel-overload environment for the onset of metabolic disease. Impaired glucose tolerance and more severe inflammation in VAT and liver only occurred in TG pigs after 12-week HFHSD feeding. Interestingly, although there was no significant difference in serum aminotransferases levels between the two groups, histological evidence of liver inflammation is clear in TG pigs. Transcriptomic data also support the histological findings in the liver. This result is similar to clinical NASH patients, indicating that serum aminotransferases in NAFLD and NASH cannot accurately reflect liver histological progress (43).

Therefore, TG pigs obtained pathogenic genetic background of human metabolic disorders, thus were more prone to developed metabolic dysfunction under energy challenge.

### Islet Deficiency and Metabolic Homeostasis Imbalance in Triple-Transgenic Pigs

Triple-transgenic pigs had strong insulin and glucagon staining in pancreatic sections. In healthy islets, paracrine insulin inhibits glucagon secretion of α-cells. However in T2DM patients, α-cell insulin resistance and hyperglucagonemia may also be observed (44). We speculate that the strong glucagon staining in TG islets might due to insulin resistance of α-cells. Interestingly, triple-transgenic pigs had increased pancreatic lipid deposition than control pigs, which we speculate lead by increased insulin content in micro-environment. Meanwhile, pancreatic adipocytes secret cytokines and release free fatty acids (FFA) that modulate the pancreas micro-environment, thus have a cross-talk with β-cells (45). The increased lipid deposition in the pancreas of TG pigs might also influence islet hormone secretion to some extent. Remarkably, the concept of nonalcoholic fatty pancreas disease has drawn attention in recent years, yet the exact pathophysiology is not fully understood (46). In this study, whether severe pancreatic lipid deposition was directly caused by transgenic effect or indirectly by systemic metabolic changes remains unclear, and the exact mechanism needs further exploration.

### NASH-Related Features in the Liver of Triple-Transgenic Pigs

Owing to the long life span, generally, few months or even years are needed to develop a pig model of metabolic syndrome by simple dietary intervention. In a DIO Gottingen pig model with 70 weeks’ high-fat/high-energy diet intervention, severe adiposity, changed serum metabolites and impaired glucose tolerance were found, and large-scale inflammation and necrosis in VAT were also observed (47). In another study, some inflammatory genes expression were changed, even down-regulated, in Gottingen pigs fed with a high fat/fructose/cholesterol diet for 13 months, suggesting “healthy fat accumulation” (48). Ossabaw pigs are outstanding models for metabolic diseases, as their thrifty genotype and miniature size makes them susceptible to metabolic syndrome. Ossabaw pigs fed with the modified atherogenic diet for 24 weeks developed a liver injury, including ballooning, steatotic KCs and fibrosis, which was close to human non-alcoholic steatohepatitis (NASH) (49). However, these pig models have not developed typical inflammatory foci in the liver.

**Figure 6:**
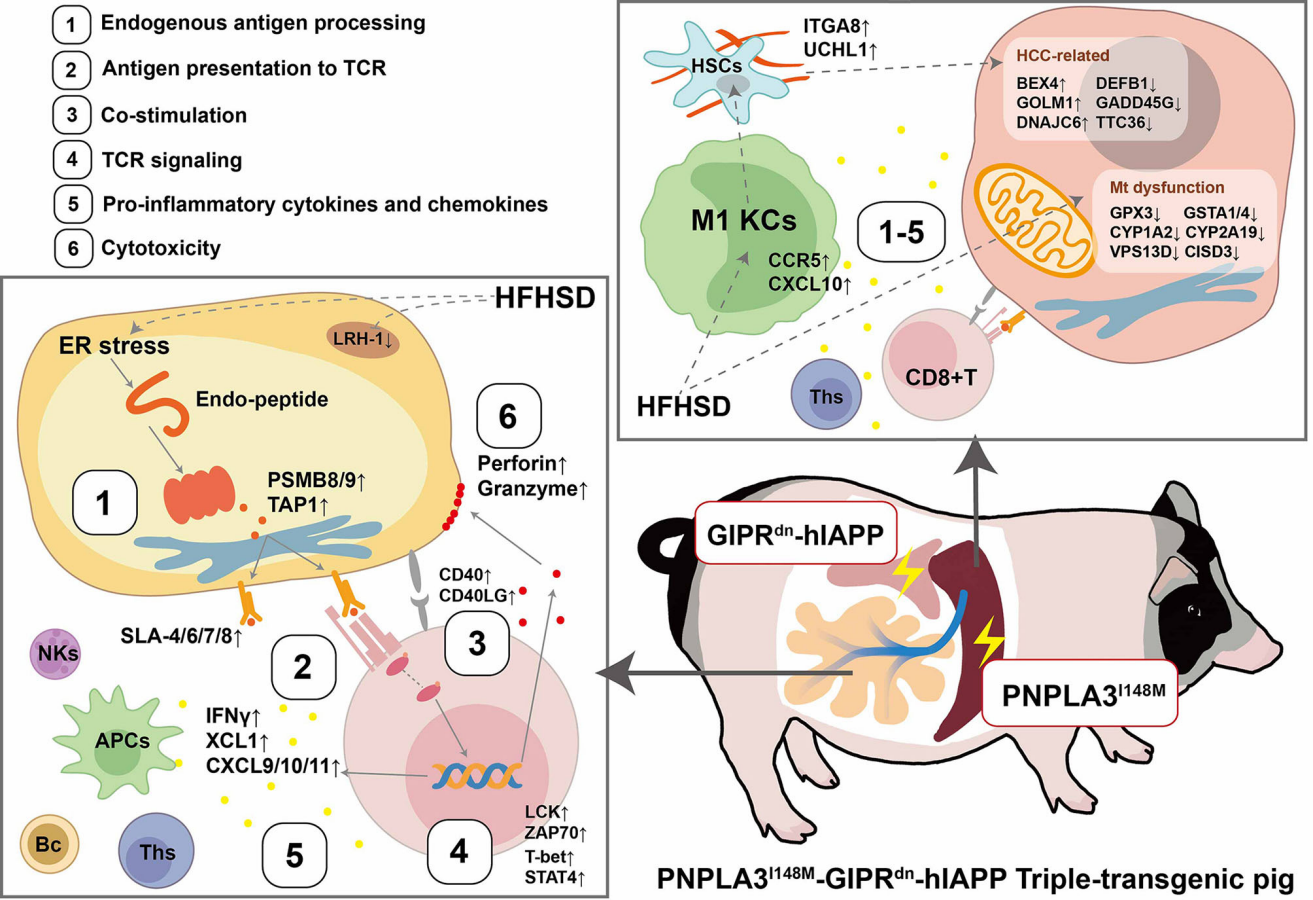
Landscape of CD8+ T cells involving in metaflammation in VAT and liver of transgenic pigs. Stressed adipocytes suffered from fuel overload, generated changed endogenous antigens, and present them through MHC-I complex (1). TCR complex on CD8^+^ T cells recognizes MHC-I antigen (2). In company with co-stimulation (3), down-stream signal was activated (4), a series of pro-inflammatory cytokines and chemokines, including IFNγ were released by CD8^+^ T cells (5), recruiting antigen-presenting cells (APCs) and CD4^+^ T helper cells (Th1). Meanwhile, effector CD8^+^ T cell secret cytotoxic molecules, including perforin and granzyme, inducing cytotoxic effects (6). Liver metabolic homeostasis was undermined by PNPLA3^I148M^ expression and over-nutrient. Inflammatory components that originated from visceral adipose tissue may mediate the crosstalk with the liver via blood circulation, and then trigger liver metaflammation.

Here, after a 12-week short-term HFHSD intervention, we see a decreased anti-oxidative ability, a decreased mitochondrial function and increased pro-fibrosis genes, suggesting metabolic stress in hepatocytes of TG pigs (**Figure 6**). Meanwhile, significant inflammatory foci were noticeable, but hepatic steatosis was not obvious at this stage. These results are in accordance with the fact that pigs are resistant to developing macrovesicular steatosis in the liver. Unlike humans and rodents, the primary site of *de novo* lipogenesis in pigs is adipose rather than the liver (49). According to the “two-hit” hypothesis of NAFLD, it is aggravated inflammation that leads to simple steatosis to riskier NASH, so the typical inflammation in the liver was the prominent feature of the triple-transgenic pig model.

TG pigs had highly functional coincidence with a detailed transcriptional network analysis on human NASH, which introduced that firstly, a NASH-linked gene signature was enriched in inflammatory responses, antigen presentation and cytotoxic cells; and secondly, hepatic CD8^+^ T cells, conventional dendritic cells and cytotoxic cell in the blood are associated with the progression from simple steatosis to NASH (50). Our work highlighted the metaflammation-related antigen presentation in transgenic pigs. On the one hand, crucial components of the MHC-I antigen-presenting complex and TCR antigen-recognizing complex were increased. Meanwhile, the downstream kinases of TCR signaling were also evaluated, which facilitate intracellular signaling and lead to lymphokine production. On the other hand, high expression of *CCR5* and *CXCL10* suggested that the liver is recruiting macrophages in TG pigs. Recruited macrophages and KCs act as professional APCs in the liver, play a key role in liver protective immunity and tolerance; when shifted to pro-inflammatory phenotype by HFD, they will recruit a series of immune cells including CD4^+^ Ths by pro-inflammatory cytokines (IL-6, TNF-α) and MHC-II antigen presentation. Hepatic stellate cells will also be stimulated by activated macrophages, eventually triggering fibrosis in NASH (51). These results illustrated that TG pigs maybe a promising NASH model, as they have highly resemblance with human NASH in a genetic, metabolic and inflammatory background.

### Link Between VAT Metabolic Stress and Inflammation

Systemic metaflammation was generally considered to be initiated from dysfunctional adipose tissue (1). Dysfunctional adipocytes were characterized by decreased insulin sensitivity, hypoxia, increased intracellular stress, autophagy, anti- or pro-inflammatory adipokines, apoptosis and limited cell expansion. The metabolic stress of adipocytes might firstly be recognized by CD8^+^ T cells *via* MHC-I antigen presentation. Then CD8^+^ T cells are activated and enhanced by co-stimulation, induce pro-inflammatory cytokine secretion and cytotoxicity, consequently attract and activate APCs, CD4^+^ Ths and other immune cells, then amplify inflammatory signals inter- and intro-tissues (**Figure 6**). Adiponectin is a classical anti-inflammatory agent, which plays an important role in mediating crosstalk between adipose tissue and other organs by reducing IFN-γ and IL-17 positive CD4^+^ Ths and dampens the differentiation of naïve T cells into pro-inflammatory Ths. Continuous lower circulation adiponectin might be one of the reasons for increased number of immune cells and up-regulated inflammatory genes in VAT of TG pigs.

Under the challenge of HFHSD, the pathological and transcriptional changes in VAT of triple-transgenic pigs were highly similar to those of obese and T2DM patients. For example, *ANGPTL4*, an adipokine that negatively regulates lipoprotein lipase activity, was increased in VAT of obese patients with abnormal glucose metabolism, insulin resistance and VAT inflammation. In diabetic patients, *ANGPTL4* is induced by FFA and hypoxia (52). Our findings are consistent with these researches that in VAT of triple-transgenic pigs with impaired glucose tolerance and heterogenic adipocytes, and *ANGPTL4* mRNA level was increased, as well as VAT inflammation. Another one is LRH-1, a nuclear receptor, participates in maintaining hepatic phospholipid balance, regulating glucose metabolism, reducing ER stress, and regulating pancreatic development (53). In this study, LRH-1 was significantly down-regulated in VAT of TG pigs, accompanied by up-regulation of a group of pro-inflammation genes. LRH-1 mitigates liver steatosis in a ligand-dependent manner, and is down-regulated in the liver of NAFL or NASH patients (53). Meanwhile, inflammatory genes were up-regulated in *NR5A2^+/−^* mice, suggesting the anti-inflammatory function of LRH-1 under homeostatic conditions in the pancreas (54). Although LRH-1 is considered a promising treatment target in both NAFLD/NASH and type 1 diabetes, few studies focus on LRH-1 function in adipose tissue. Further verification of how LRH-1 contributes to adipose meta-homeostasis and inflammation is still needed, here we suggested that LRH-1 is a node connecting metabolic stress and inflammation in VAT, and again emphasize its role in treating metabolic diseases.

### Conclusions and Future Perspectives

This study reports the basic physiology, histology, and transcriptomic characteristics of *PNPLA3^I148M^-GIPR^dn^-hIAPP* triple-transgenic Bama pigs. The short-term dietary intervention of these pigs proved that they are less capable of maintaining metabolic homeostasis under excessive energy challenges. Clear evidence of metaflammation in the liver and VAT suggested that TG pigs are a promising model for metabolic diseases, including but not limited to NASH and T2DM. The initiation of tissue metaflammation is exhibited at this stage, but hepatic steatosis, hepatitis and fibrosis are long-term processes. Therefore, to comprehensively evaluate the triple-transgenic pig model’s reliability in medical research, longer-term studies on TG pigs are underway.

Combining transcriptome profiles in VAT and liver, we found some shared genes that function in antigen processing and presentation, TCR signaling, and co-stimulatory molecules. Since co-stimulatory molecules are prospective therapeutic targets for metaflammation, TG pigs with the same co-stimulation feature as human obesity are promising animal models in drug exploration. Meanwhile, the greater omentum has direct impact on liver metabolism through the portal system. We suspect that CD8^+^ T cell activation in VAT stimulated pro-inflammatory cytokine and chemokine production, which may consequently secrete into the portal system and promote liver inflammation (**Figure 6**). To better understand the mechanism of obesity-related metaflammation and the contribution of tissue immune-crosstalk in metabolic diseases, further research is needed. Proteome and metabolome analysis on a blood sample collected from the portal vein and hepatic vein in the procession of metaflammation will help in understanding the molecular influence from the intestine, pancreas, and VAT on NASH development.

RNA-seq data at tissue level indicated activation of CD8^+^ T cells. However, the dynamic change of immune cell population in metaflammation was unclear, because the expression of many immune genes is not restricted to a single cell group. For example, MHC-II molecules could be found on professional APCs and tissue cells, perforin and granzyme could be secreted by NKs, and cytotoxic CD8^+^ T cells, *LCK* and *ZAP70* are important kinases in CD8^+^ and CD4^+^ TCR signaling, so we could not determine which population play the dominant role. Meanwhile, immune cells themselves sense nutrient changes in the microenvironment, the metabolic status of immune cells are also an important determinant of tissue and systemic homeostasis (3). Single-cell RNA sequencing (scRNA-seq) is a promising technique that provides us solutions. Triple-transgenic Bama pig is the first model that displays systemic metaflammation. Using biopsy samples of triple-transgenic pigs at different time point of disease progression, we would like to investigate the initiation of tissue metaflammation, the action and function of each immune cell group under energy-stress, the interaction between immune cells and stromal components, and the crosstalk between metabolic tissues.

It was well-proven that infiltration of CD8^+^ T cells in metabolic tissues is the precondition of macrophage recruitment and obesity-induced chronic inflammation (4), but how does metabolic stress induce MHC-I associated immunopeptide generation? What is the source of these endogenous antigens? Simultaneous enhancement of ribosome and proteasome functions indicated that these steps might link metabolic induced endoplasmic reticulum stress, miss-folding protein degradation and endogenous antigen processing. Further study on the newly reported MHC-Ia molecular *SLA-4* is also needed to confirm its protein expression, structure and antigen-presenting capability.

## Data Availability Statement

The datasets presented in this study can be found in online repositories. Sequencing data generated in this study were deposited in Sequence Read Archive (SRA) (https://trace.ncbi.nlm.nih.gov/Traces/sra/) under the accession number PRJNA686141 and PRJNA686271.

## Ethics Statement

The animal study was reviewed and approved by Animal Care and Use Committee of the Germplasm Resource Center of Chinese Experimental Minipigs Institute of Animal Sciences, Chinese Academy of Agricultural Sciences.

## Author Contributions

KZ performed the experiments, data analyses, data visualization, and wrote the manuscript. CT and JPX facilitated sample collection and performed the experiments. JR, JHX, WZ LX, HY, NX, BX, CL, and TW participated in the construction of transgenic animals. YW and MS provided conceptual insights and edited the manuscript. XX, JF, and YS supervised the project and provided financial support. All authors contributed to the article and approved the submitted version.

## Funding

This study was supported by the National Natural Science Foundation of China (Grant NO. 81770832, 32070535), The National Science and Technology Major Project (Grant NO. 2016ZX08010-003), and the Agricultural Science and Technology Innovation Program (Grant NO. ASTIP-IAS05).

## Conflict of Interest

The authors declare that the research was conducted in the absence of any commercial or financial relationships that could be construed as a potential conflict of interest.
