# Supplementary material for: CD8+ T Cells Involved in Metabolic Inflammation in Visceral Adipose Tissue and Liver of Transgenic Pigs
Source: Front Immunol. 2021 Jul 12;12:690069. doi: 10.3389/fimmu.2021.690069 (PMC8311854; doi:10.3389/fimmu.2021.690069)
Supplement: Supplementary file 1 [file DataSheet_1.docx]

Supplementary Material

**
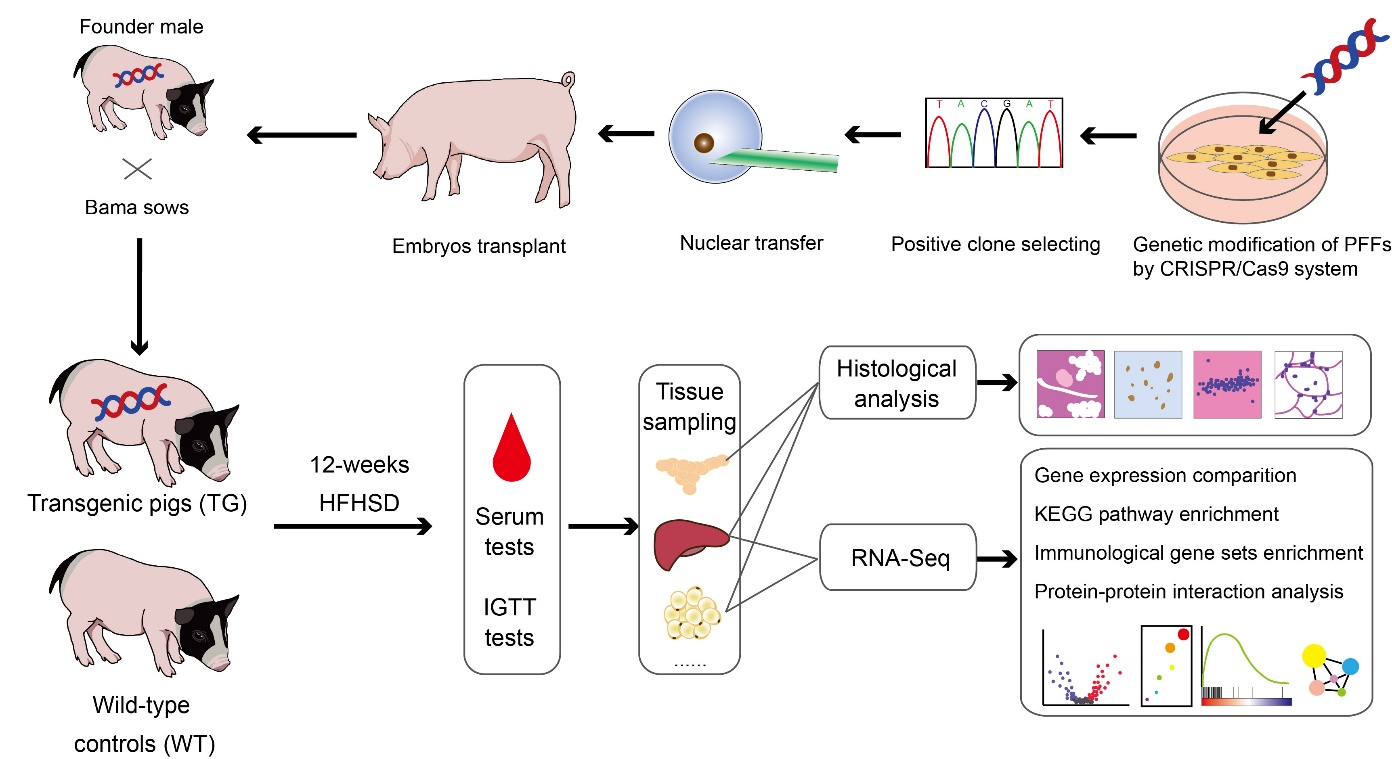
**

**Supplementary Figure 1: Schematics of the study design.** To access genetic modification, the vector carrying PNPLA3^I148M^-GIPR^dn^-hIAPP target sequences and the vector carrying CRISPR/Cas9 system were transfected into porcine fetal fibroblasts. Positive clones were selected by DNA sequencing, then used for nuclear transfer and embryos transplant. One transgenic positive founder male was obtained and mated with wild-type Bama sows to established the experimental group. 10 transgenic male pigs and 10 matched wild-type control pigs were used in this study. After fed with HFHSD for 12 weeks, serum indicators and intravenous glucose tolerance were tested; tissues were carefully collected to construct a bio-sample bank. Histological analysis and/or RNA-seq analysis were performed on pancreas, liver, VAT and subcutaneous adipose tissue.


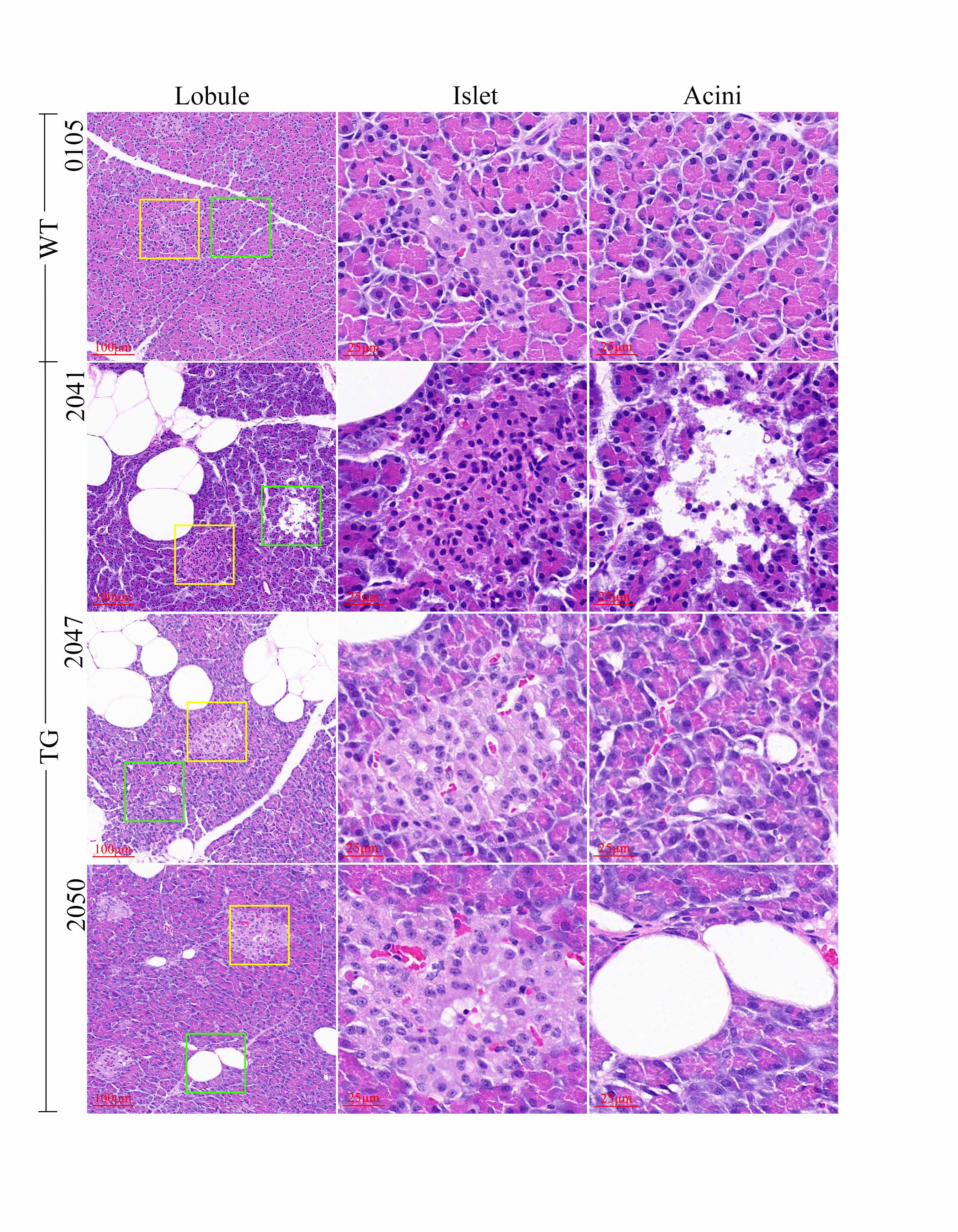


**Supplementary Figure 2: HE staining of pancreas.** Scale bars are 50 μm in 2× magnified view and 500 μm in 20× magnified view.

**
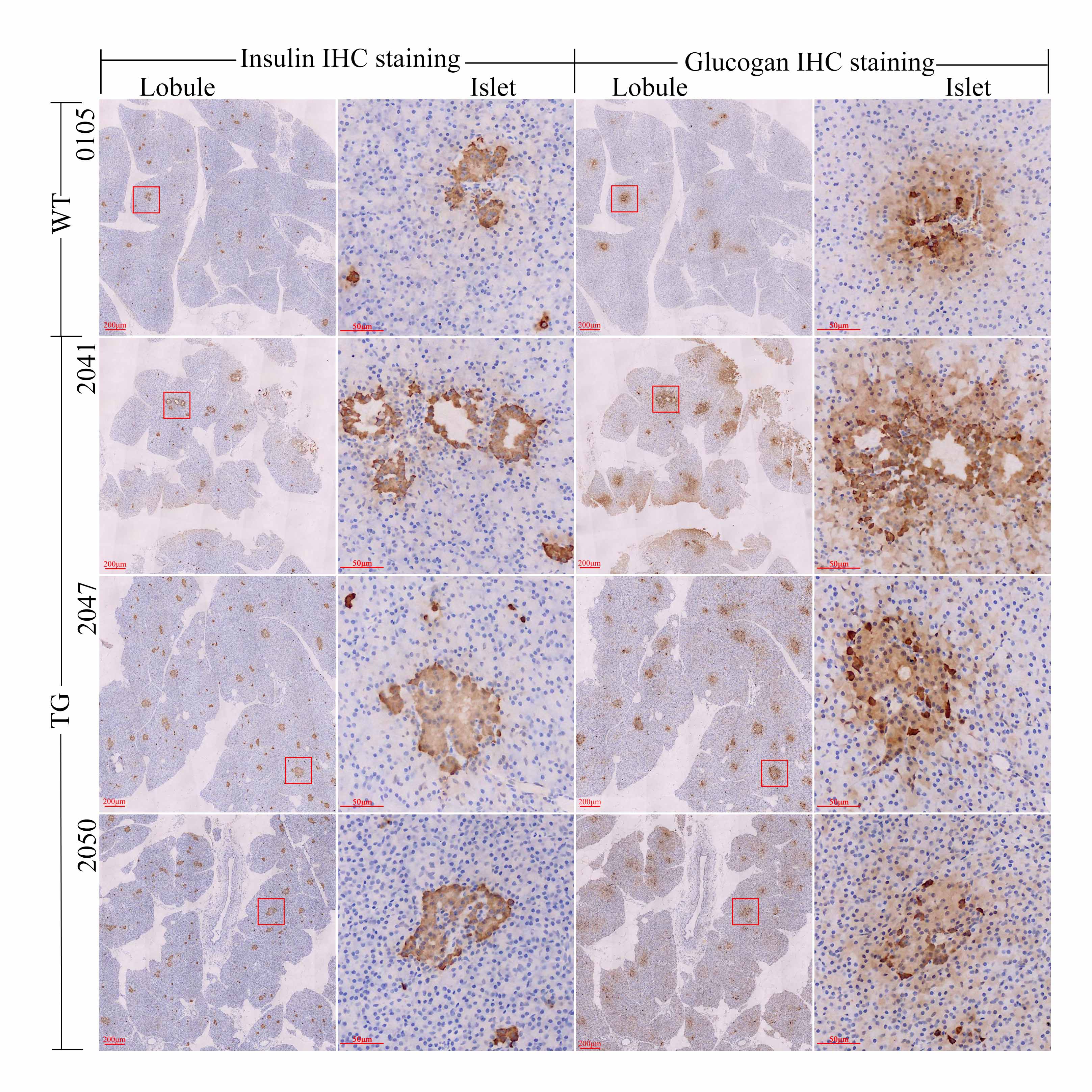
**

**Supplementary Figure 3: Insulin and glucagon staining of pancreas.** Immunohistochemical staining of insulin and glucagon were conducted in two continuous sections. Islets were pointed out by red boxes and presented in the second and the forth column. Scale bars are 200 μm in lobule views and 50 μm in islet views.


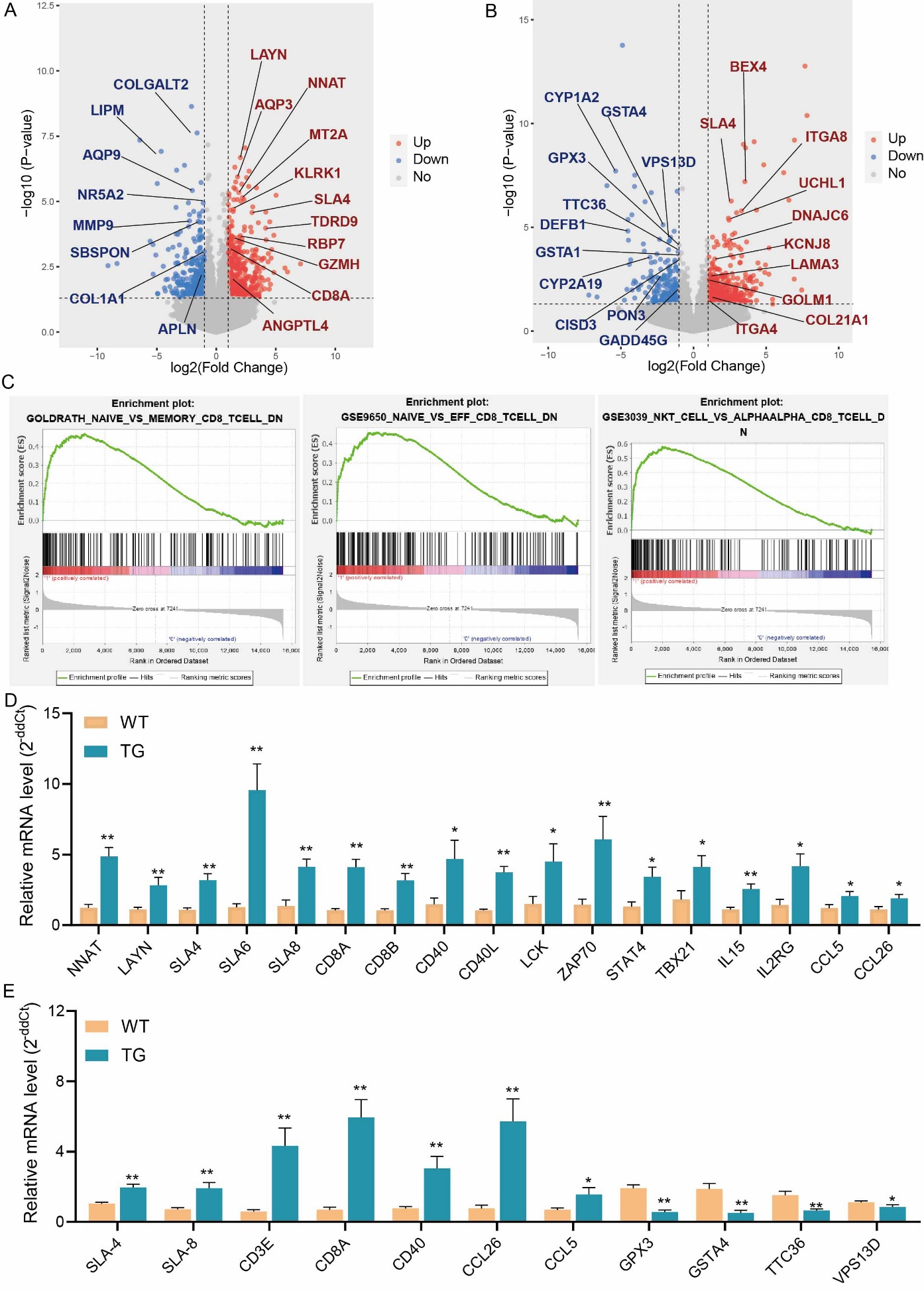


**Supplementary Figure 4: RT-QPCR results and RNA-seq data.** (**A-B**) Volcano map of VAT (A) and liver (B). Differentially expressed genes were filtered out by the threshold P<0.05 and |log2 (Fold change)|> 1. (**C**) GSEA enrichment plot map of crucial immunological gene sets in VAT. (**D-E**) RT-QPCR results of important core enrichment genes in VAT (D) and liver (E). Relative mRNA level were calculated using 2-^△△^ct method, n= 5 per group. Data are mean ± SEM; statistical difference was tested using Student’s t-test with Bonferroni correction; *P<0.05, **P<0.01.

**Supplementary Table 1. Serum data**

|  | WT (n=10) | TG (n=10) | *P*-value |
| --- | --- | --- | --- |
| Glucose (mmol/L) | 6.03±0.69 | 5.47±0.9 | 0.1452 |
| Insulin (μIU/mL) | 64.31±13.02 | 61.95±10.60 | 0.6622 |
| Glucagon (pg/mL) | 81.23±10.01 | 83.37±16.63 | 0.7315 |
| TC (mmol/L) | 2.59±0.45 | 2.38±0.47 | 0.3246 |
| LDL-C (mmol/L) | 0.72±0.16 | 0.67±0.19 | 0.4877 |
| HDL-C (mmol/L) | 1.35±0.28 | 1.10±0.16 | 0.0225 |
| Triglycerides (mmol/L) | 0.38±0.18 | 0.41±0.09 | 0.6514 |
| NEFA (μmol/L) | 61.51±24.21 | 80.90±21.74 | 0.0758 |
| ALT (U/L) | 25.16±13.9 | 24.22±5.31 | 0.8439 |
| AST (U/L) | 23.17±11.46 | 21.27±9.75 | 0.6943 |
| Leptin (pg/mL) | 714.04±146.22 | 661.4±164.21 | 0.4588 |
| Adiponectin (ng/mL) | 1102.48±293.89 | 782.49±81.67 | 0.0038 |
| CRP (ng/mL) | 832.71±236.42 | 894.47±222.01 | 0.5546 |

Student’s t-test was used for statistical analysis.

**Supplementary Table 2. KEGG pathway enrichment by GSEA.**

| **KEGG Pathways** | **Size** | **ES** | **NES** | **NOM p-value** | **FDR q-value** | **Rank at Max** |
| --- | --- | --- | --- | --- | --- | --- |
| **Up-regulated in TG VAT** |  |  |  |  |  |  |
| Ribosome | 58 | 0.75 | 2.78 | 0.0000 | 0.0000 | 2856 |
| Primary immunodeficiency | 30 | 0.77 | 2.45 | 0.0000 | 0.0000 | 1593 |
| Proteasome | 40 | 0.64 | 2.18 | 0.0000 | 0.0000 | 4672 |
| T cell receptor signaling pathway | 99 | 0.53 | 2.16 | 0.0000 | 0.0000 | 2653 |
| Allograft rejection | 17 | 0.78 | 2.15 | 0.0000 | 0.0003 | 2049 |
| Type I diabetes mellitus | 22 | 0.70 | 2.08 | 0.0000 | 0.0006 | 2261 |
| Autoimmune thyroid disease | 20 | 0.67 | 1.92 | 0.0020 | 0.0048 | 1142 |
| Intestinal immune network for IgA production | 31 | 0.58 | 1.90 | 0.0000 | 0.0051 | 1379 |
| Natural killer cell mediated cytotoxicity | 76 | 0.45 | 1.75 | 0.0000 | 0.0284 | 3159 |
| Systemic lupus erythematosus | 36 | 0.50 | 1.65 | 0.0061 | 0.0686 | 2049 |
| Antigen processing and presentation | 35 | 0.49 | 1.60 | 0.0164 | 0.0939 | 2193 |
| Tryptophan metabolism | 35 | 0.49 | 1.60 | 0.0165 | 0.0880 | 1850 |
| Toll like receptor signaling pathway | 79 | 0.41 | 1.60 | 0.0150 | 0.0813 | 3322 |
| Hematopoietic cell lineage | 64 | 0.43 | 1.58 | 0.0123 | 0.0929 | 2194 |
| Retinol metabolism | 25 | 0.51 | 1.55 | 0.0121 | 0.1058 | 3036 |
| Cell adhesion molecules (CAMs) | 102 | 0.38 | 1.55 | 0.0063 | 0.1019 | 2186 |
| Cytosolic DNA sensing pathway | 37 | 0.46 | 1.54 | 0.0209 | 0.0997 | 2081 |
| Spliceosome | 105 | 0.37 | 1.51 | 0.0105 | 0.1255 | 4421 |
| Drug metabolism other enzymes | 21 | 0.51 | 1.48 | 0.0415 | 0.1440 | 2700 |
| Chemokine signaling pathway | 145 | 0.34 | 1.48 | 0.0000 | 0.1382 | 3159 |
| Cytokine-cytokine receptor interaction | 191 | 0.33 | 1.47 | 0.0020 | 0.1431 | 2128 |
| Fatty acid metabolism | 32 | 0.46 | 1.46 | 0.0482 | 0.1451 | 1151 |
| **Down-regulated in TG VAT** |  |  |  |  |  |  |
| Steroid biosynthesis | 16 | -0.67 | -1.80 | 0.0019 | 0.0786 | 4023 |
| **Up-regulated in TG liver** |  |  |  |  |  |  |
| Natural killer cell mediated cytotoxicity | 76 | 0.59 | 2.10 | 0.0000 | 0.0020 | 3090 |
| Fc epsilon RI signaling pathway | 71 | 0.58 | 2.04 | 0.0000 | 0.0010 | 2643 |
| Primary immunodeficiency | 30 | 0.65 | 1.94 | 0.0000 | 0.0036 | 610 |
| T-cell receptor signaling pathway | 99 | 0.52 | 1.93 | 0.0000 | 0.0029 | 3236 |
| Asthma | 15 | 0.74 | 1.90 | 0.0000 | 0.0040 | 602 |
| Cell adhesion molecules (CAMs) | 102 | 0.51 | 1.88 | 0.0000 | 0.0037 | 2091 |
| Focal adhesion | 177 | 0.47 | 1.86 | 0.0000 | 0.0042 | 3090 |
| GnRH signaling pathway | 87 | 0.51 | 1.86 | 0.0000 | 0.0044 | 4148 |
| Allograft rejection | 17 | 0.69 | 1.85 | 0.0000 | 0.0040 | 1484 |
| Glioma | 55 | 0.54 | 1.84 | 0.0014 | 0.0047 | 3090 |
| Calcium signaling pathway | 155 | 0.46 | 1.81 | 0.0000 | 0.0068 | 2794 |
| Chemokine signaling pathway | 145 | 0.47 | 1.80 | 0.0000 | 0.0070 | 3975 |
| B-cell receptor signaling pathway | 63 | 0.52 | 1.79 | 0.0000 | 0.0067 | 3731 |
| ECM receptor interaction | 77 | 0.50 | 1.77 | 0.0013 | 0.0089 | 2617 |
| Chronic myeloid leukemia | 67 | 0.51 | 1.76 | 0.0000 | 0.0088 | 3433 |
| Aldosterone regulated sodium reabsorption | 35 | 0.57 | 1.76 | 0.0028 | 0.0085 | 2620 |
| Pathways in cancer | 286 | 0.42 | 1.75 | 0.0000 | 0.0088 | 3731 |
| Neurotrophin signaling pathway | 113 | 0.46 | 1.74 | 0.0000 | 0.0099 | 3635 |
| Melanoma | 64 | 0.50 | 1.73 | 0.0000 | 0.0100 | 3090 |
| Axon guidance | 121 | 0.45 | 1.72 | 0.0000 | 0.0112 | 2856 |
| Type II diabetes mellitus | 41 | 0.54 | 1.72 | 0.0013 | 0.0111 | 3628 |
| ErbB signaling pathway | 79 | 0.48 | 1.72 | 0.0000 | 0.0106 | 3693 |
| Prostate cancer | 80 | 0.48 | 1.72 | 0.0000 | 0.0102 | 3280 |
| Long term depression | 63 | 0.50 | 1.71 | 0.0013 | 0.0107 | 4148 |
| Phosphatidylinositol signaling system | 67 | 0.48 | 1.70 | 0.0013 | 0.0114 | 3823 |
| Long term potentiation | 61 | 0.49 | 1.68 | 0.0013 | 0.0142 | 3758 |
| Progesterone mediated oocyte maturation | 76 | 0.47 | 1.66 | 0.0013 | 0.0159 | 3646 |
| Gap junction | 79 | 0.46 | 1.66 | 0.0000 | 0.0167 | 2899 |
| Hematopoietic cell lineage | 64 | 0.48 | 1.65 | 0.0054 | 0.0175 | 2698 |
| Ether lipid metabolism | 29 | 0.56 | 1.65 | 0.0160 | 0.0174 | 2058 |
| Renal cell carcinoma | 57 | 0.49 | 1.64 | 0.0040 | 0.0197 | 3273 |
| MAPK signaling pathway | 240 | 0.40 | 1.63 | 0.0000 | 0.0192 | 4261 |
| Notch signaling pathway | 46 | 0.51 | 1.63 | 0.0053 | 0.0188 | 3289 |
| Fc gamma R mediated phagocytosis | 83 | 0.46 | 1.62 | 0.0025 | 0.0203 | 2620 |
| Intestinal immune network for IgA production | 31 | 0.54 | 1.61 | 0.0130 | 0.0231 | 1178 |
| Leishmania infection | 52 | 0.49 | 1.61 | 0.0095 | 0.0229 | 3645 |
| Leukocyte transendothelial migration | 106 | 0.43 | 1.61 | 0.0013 | 0.0223 | 3241 |
| Dilated cardiomyopathy | 78 | 0.45 | 1.60 | 0.0013 | 0.0227 | 2646 |
| Non-small cell lung cancer | 49 | 0.49 | 1.60 | 0.0108 | 0.0240 | 3090 |
| Cytokine-cytokine receptor interaction | 191 | 0.40 | 1.59 | 0.0000 | 0.0251 | 3003 |
| Small cell lung cancer | 71 | 0.45 | 1.58 | 0.0078 | 0.0266 | 3146 |
| VEGF signaling pathway | 64 | 0.45 | 1.57 | 0.0067 | 0.0284 | 4233 |
| Adherens junction | 65 | 0.45 | 1.56 | 0.0105 | 0.0314 | 5245 |
| Neuroactive ligand receptor interaction | 235 | 0.38 | 1.56 | 0.0000 | 0.0319 | 2925 |
| Autoimmune thyroid disease | 20 | 0.57 | 1.55 | 0.0332 | 0.0314 | 329 |
| JAK-STAT signaling pathway | 119 | 0.41 | 1.54 | 0.0013 | 0.0347 | 3693 |
| Inositol phosphate metabolism | 49 | 0.47 | 1.53 | 0.0137 | 0.0392 | 3823 |
| Endometrial cancer | 45 | 0.46 | 1.51 | 0.0139 | 0.0467 | 3090 |
| Viral myocarditis | 40 | 0.48 | 1.50 | 0.0362 | 0.0489 | 3961 |
| Epithelial cell signaling in helicobacter pylori infection | 59 | 0.43 | 1.50 | 0.0202 | 0.0490 | 3984 |
| Vascular smooth muscle contraction | 99 | 0.41 | 1.49 | 0.0100 | 0.0495 | 4148 |
| Regulation of actin cytoskeleton | 191 | 0.37 | 1.48 | 0.0023 | 0.0528 | 3090 |
| TGF beta signaling pathway | 81 | 0.41 | 1.48 | 0.0078 | 0.0556 | 3358 |
| Pancreatic cancer | 61 | 0.42 | 1.46 | 0.0171 | 0.0630 | 4360 |
| Antigen processing and presentation | 35 | 0.46 | 1.45 | 0.0498 | 0.0680 | 4591 |
| Melanogenesis | 92 | 0.40 | 1.44 | 0.0205 | 0.0742 | 2774 |
| Bladder cancer | 37 | 0.46 | 1.43 | 0.0478 | 0.0775 | 2643 |
| Systemic lupus erythematosus | 36 | 0.45 | 1.42 | 0.0403 | 0.0803 | 1538 |
| Endocytosis | 154 | 0.37 | 1.42 | 0.0121 | 0.0797 | 4149 |
| Hypertrophic cardiomyopathy (HCM) | 74 | 0.40 | 1.40 | 0.0288 | 0.0915 | 3152 |
| Toll-like receptor signaling pathway | 79 | 0.39 | 1.39 | 0.0231 | 0.0948 | 3645 |
| Oocyte meiosis | 94 | 0.37 | 1.39 | 0.0346 | 0.0924 | 3895 |
| Cell cycle | 111 | 0.36 | 1.36 | 0.0335 | 0.1152 | 3378 |
| Olfactory transduction | 121 | 0.35 | 1.34 | 0.0357 | 0.1339 | 1585 |
| **Down-regulated in TG liver** |  |  |  |  |  |  |
| Ribosome | 58 | -0.76 | -2.97 | 0.0000 | 0.0000 | 3001 |
| Oxidative phosphorylation | 93 | -0.64 | -2.69 | 0.0000 | 0.0000 | 3313 |
| Parkinsons disease | 91 | -0.58 | -2.44 | 0.0000 | 0.0000 | 3313 |
| Huntingtons disease | 141 | -0.47 | -2.17 | 0.0000 | 0.0005 | 3313 |
| Biosynthesis of unsaturated fatty acids | 19 | -0.68 | -2.07 | 0.0000 | 0.0021 | 2650 |
| Alzheimers disease | 123 | -0.44 | -1.95 | 0.0000 | 0.0050 | 1702 |
| Glutathione metabolism | 37 | -0.54 | -1.91 | 0.0000 | 0.0077 | 1578 |
| Drug metabolism cytochrome p450 | 25 | -0.54 | -1.76 | 0.0000 | 0.0257 | 1569 |
| Metabolism of xenobiotics by cytochrome p450 | 25 | -0.50 | -1.66 | 0.0117 | 0.0485 | 1958 |
| Citrate cycle TCA cycle | 28 | -0.48 | -1.59 | 0.0176 | 0.0695 | 1957 |
| RNA polymerase | 23 | -0.49 | -1.55 | 0.0245 | 0.0824 | 1430 |
| Proteasome | 40 | -0.40 | -1.45 | 0.0355 | 0.1560 | 4490 |

**Supplementary Table 3. Immunological gene sets enrichment by GSEA.**

| **Gene set ID** | **Brief description** | **Size** | **ES** | **NES** | **NOM**  **p-value** | **FDR**  **q-value** | **RANK at MAX** |
| --- | --- | --- | --- | --- | --- | --- | --- |
| **Up-regulated in TG VAT (Top 20)** | |  |  |  |  |  |  |
| GSE7218_UNSTIM_VS_ANTIGEN_STIM_THROUGH_IGG_BCELL_DN | Genes down-regulated in B lymphocytes: expressing IgM BCR fusion and untreated versus expressing IgMG BCR fusion and treated by anti-HEL. | 133 | 0.62 | 2.61 | 0.0000 | 0.0000 | 2527 |
| GSE3039_NKT_CELL_VS_ALPHAALPHA_CD8_TCELL_DN | Genes down-regulated in NKT cells versus CD8A T cells. | 163 | 0.58 | 2.50 | 0.0000 | 0.0000 | 1987 |
| GSE7218_IGM_VS_IGG_SIGNAL_  THGOUGH_ANTIGEN_BCELL_DN | Genes down-regulated in B lymphocytes treated by anti-HEL and expressing BCR fusions with: IgM versus IgMG. | 148 | 0.57 | 2.42 | 0.0000 | 0.0000 | 1986 |
| GSE7509_UNSTIM_VS_FCGRIIB_STIM_  DC_DN | Genes down-regulated in dendritic cells: untreated versus anti-FcgRIIB. | 151 | 0.54 | 2.32 | 0.0000 | 0.0000 | 1967 |
| GSE22886_NAIVE_TCELL_VS_DC_UP | Genes up-regulated in comparison of naive CD4 CD8 T cells versus unstimulated dendritic cells (DC). | 152 | 0.54 | 2.31 | 0.0000 | 0.0000 | 2717 |
| GSE2405_0H_VS_9H_A_PHAGOCYTOPHILUM_STIM_NEUTROPHIL_DN | Genes down-regulated in polymorphonuclear leukocytes (9h): control versus infection by A. phagocytophilum. | 160 | 0.53 | 2.31 | 0.0000 | 0.0000 | 3948 |
| GSE42088_UNINF_VS_LEISHMANIA_  INF_DC_4H_DN | Genes down-regulated in dendritic cells: untreated versus 4h after infection of Leishmania major. | 155 | 0.52 | 2.23 | 0.0000 | 0.0000 | 2861 |
| GSE2405_0H_VS_24H_A_PHAGOCYTOPHILUM_STIM_NEUTROPHIL_UP | Genes up-regulated in polymorphonuclear leukocytes (24h): control versus infection by A. phagocytophilum. | 152 | 0.51 | 2.21 | 0.0000 | 0.0000 | 2740 |
| GSE7509_UNSTIM_VS_IFNA_STIM_  IMMATURE_DC_DN | Genes down-regulated in immature dendritic cells: untreated versus interferon alpha. | 154 | 0.50 | 2.18 | 0.0000 | 0.0000 | 1986 |
| GSE22886_NAIVE_CD8_TCELL_VS_  MONOCYTE_UP | Genes up-regulated in comparison of naive CD8 T cells versus day 0 monocytes. | 161 | 0.49 | 2.13 | 0.0000 | 0.0000 | 1443 |
| GSE45739_UNSTIM_VS_ACD3_ACD28_  STIM_WT_CD4_TCELL_DN | Genes down-regulated in CD4 T cells: unstimulated versus activated. | 164 | 0.48 | 2.10 | 0.0000 | 0.0000 | 2524 |
| GSE9006_TYPE_1_VS_TYPE_2_DIABETES_PBMC_AT_DX_UP | Genes up-regulated in peripheral blood mononuclear cells (PBMC) from patients with type 1 diabetes at the time of diagnosis versus those with type 2 diabetes at the time of diagnosis. | 180 | 0.47 | 2.06 | 0.0000 | 0.0000 | 4521 |
| GOLDRATH_NAIVE_VS_MEMORY_CD8_TCELL_DN | Genes down-regulated in comparison of naive CD8 T cells versus memory CD8 T cells. | 167 | 0.47 | 2.04 | 0.0000 | 0.0000 | 2684 |
| GSE14000_TRANSLATED_RNA_VS_  MRNA_DC_DN | Genes down-regulated in comparison of polysome bound (translated) mRNA versus total mRNA in dendritic cells. | 133 | 0.48 | 2.04 | 0.0000 | 0.0000 | 4211 |
| GSE6259_DEC205_POS_DC_VS_CD8_  TCELL_UP | Genes up-regulated in splenic DEC205+ dendritic cells versus CD8 T cells. | 158 | 0.46 | 2.02 | 0.0000 | 0.0000 | 3477 |
| GSE37301_HEMATOPOIETIC_STEM_  CELL_VS_PRO_BCELL_DN | Genes down-regulated in hematopoietic stem cells versus pro-B cells. | 149 | 0.47 | 1.99 | 0.0000 | 0.0000 | 3383 |
| GSE9650_NAIVE_VS_EFF_CD8_TCELL_  DN | Genes down-regulated in comparison of naive CD8 T cells versus effector CD8 T cells. | 170 | 0.46 | 1.98 | 0.0000 | 0.0000 | 2682 |
| GSE22886_NAIVE_CD4_TCELL_VS_  MONOCYTE_UP | Genes up-regulated in comparison of naive CD4 T cells versus day 0 monocytes. | 160 | 0.46 | 1.98 | 0.0000 | 0.0000 | 1434 |
| GSE42088_UNINF_VS_LEISHMANIA_INF_DC_2H_DN | Genes down-regulated in dendritic cells: untreated versus 2h after infection of Leishmania major. | 158 | 0.46 | 1.97 | 0.0000 | 0.0000 | 2856 |
| GSE7460_CD8_TCELL_VS_CD4_TCELL_  ACT_UP | Genes up-regulated in comparison of ActCD8 versus ActCD4. | 177 | 0.44 | 1.97 | 0.0000 | 0.0000 | 1718 |

**Supplementary Table 4. Protein sequence alignment between species.**

| Gene Name | Uniprot Entry | | | | Alignment with human (%) | | | Similarity  * | VAT^#^ | | Liver^#^ | |
| --- | --- | --- | --- | --- | --- | --- | --- | --- | --- | --- | --- | --- |
|  | Human | Pig | Mouse | Rat | Pig | Mouse | Rat |  | Log2  FC | P-value | Log2  FC | *P*-value |
| IFNG | P01579 | P17803 | P0580 | P01581 | 60 | 38 | 36 | A | 1.25 | 0.034 | 1.61 | 0.337 |
| IL12RB1 | P42701 | F1S921 | Q60837 | E9PSU7 | 64 | 47 | 46 | A | 0.79 | 0.046 | 0.43 | 0.407 |
| IL12RB2 | Q99665 | Q8MJS1 | Q60837 | F1LRH7 | 79 | 65 | 64 | A | 1.25 | 0.006 | 0.87 | 0.084 |
| IL15 | P40933 | K7GNZ5 | P48346 | P97604 | 71 | 73 | 72 | B | 0.60 | 0.028 | -0.04 | 0.883 |
| IL15RA | Q13261 | I3LM29 | Q60819 | F1M293 | 40 | 51 | 48 | B | 0.44 | 0.042 | 0.22 | 0.651 |
| IL2 | P60568 | P26891 | P04351 | P17108 | 71 | 57 | 66 | A | 0.04 | 0.978 | -0.74 | 0.329 |
| IL2RA | P01589 | O02733 | P01590 | P26897 | 54 | 60 | 61 | C | 0.46 | 0.088 | 0.93 | 0.142 |
| IL2RG | P31785 | F6PZ59 | P34902 | Q68FU6 | 77 | 71 | 74 | A | 0.78 | 0.038 | 0.38 | 0.145 |
| CXCL10 | P02778 | Q5S1S3 | P17515 | P48973 | 80 | 68 | 68 | A | 1.32 | 0.003 | 0.91 | 0.021 |
| CXCL9 | Q07325 | B0FYK2 | P18340 | Q8K4B1 | 75 | 67 | 63 | A | 1.80 | 0.000 | 0.23 | 0.656 |
| CXCL11 | O14625 | F1RYT7 | Q9JHH5 | Q7TNL0 | 76 | 64 | 60 | A | 1.87 | 0.001 | -3.33 | 0.000 |
| CXCR3 | P49682 | K7GML5 | O88410 | Q9JII9 | 88 | 86 | 86 | A | 1.22 | 0.001 | 0.93 | 0.200 |
| CCL5 | P13501 | Q2EN89 | P30882 | P50231 | 84 | 80 | 78 | A | 1.03 | 0.001 | 0.16 | 0.584 |
| CCL26 | Q9Y258 | A0A287BN00 | F8VQM2 | D3ZQG2 | 65 | 49 | 49 | A | 1.25 | 0.005 | 1.70 | 0.003 |
| CD3D | P04234 | Q764N2 | P04235 | P19377 | 70 | 64 | 68 | A | 0.99 | 0.002 | 0.83 | 0.051 |
| CD3E | P07766 | Q7YRN2 | P22646 | D4A5M2 | 62 | 58 | 58 | A | 1.23 | 0.000 | 0.75 | 0.036 |
| CD8A | P01732 | A0A287A9Y5 | P01731 | P07725 | 60 | 45 | 48 | A | 1.19 | 0.001 | 1.01 | 0.007 |
| CD8B | P10966 | F1SVD7 | P10300 | P05541 | 48 | 50 | 54 | C | 1.61 | 0.000 | 0.61 | 0.227 |
| CD40 | P25942 | Q8SQ34 | P27512 | Q4QQW2 | 73 | 58 | 56 | A | 0.48 | 0.037 | 0.96 | 0.000 |
| CD40LG | P29965 | Q95MQ5 | P27548 | Q9Z2V2 | 86 | 77 | 77 | A | 0.95 | 0.012 | 1.27 | 0.086 |
| ICOS | Q9Y6W8 | Q9R1T7 | Q9WVS0 | Q9R1T7 | 70 | 69 | 67 | A | 0.94 | 0.015 | 0.34 | 0.519 |
| TNFRSF4 | P43489 | A0A287A7B3 | P47741 | P15725 | 65 | 61 | 60 | A | 1.43 | 0.047 | 0.98 | 0.333 |
| CD80 | P33681 | Q9BE99 | Q00609 | G3V671 | 55 | 40 | 42 | A | 0.28 | 0.075 | -0.31 | 0.350 |
| PSMB8 | P28062 | A5D9J4 | P28063 | P28064 | 90 | 88 | 89 | A | 0.42 | 0.031 | 0.16 | 0.562 |
| PSMB9 | P28065 | Q2PYM7 | P28076 | P28077 | 95 | 89 | 89 | A | 0.64 | 0.001 | 0.41 | 0.120 |
| TAP1 | Q03518 | A5D9J3 | P21958 | P36370 | 74 | 65 | 65 | A | 0.58 | 0.003 | 0.61 | 0.001 |
| LCK | P06239 | F1SV94 | P06240 | Q01621 | 96 | 97 | 97 | B | 1.00 | 0.003 | 0.66 | 0.019 |
| ZAP70 | P43403 | A0A5K1V762 | P34304 | A0A0R4J8U1 | 93 | 94 | 93 | B | 1.09 | 0.000 | 1.10 | 0.004 |
| STAT4 | Q14765 | E1U8C4 | P42228 | Q66HB2 | 97 | 95 | 95 | A | 0.73 | 0.049 | 0.95 | 0.040 |
| TBX21 | Q9UL17 | F1RWJ2 | Q9JKD8 | D3ZCM2 | 94 | 87 | 86 | A | 0.85 | 0.009 | 0.48 | 0.232 |

*Similarity: A means highest conservation between human and pig, B mean highest conservation between human and mouse, C means highest conservation between human and rat. #RNA-seq data of transgenic pigs(TG) and non-transgenic pigs(WT), log2FC were calculated by TG vs. WT. Sequence data were obtained from https://www.uniprot.org/

**Supplementary Table 5. Primers for genotyping and RT-QPCR**

| Target | Forward primer (5’-3’) | Reverse primer (5’-3’) |
| --- | --- | --- |
| H11-Negative | TGCTGGACTTCCACCGTTGT | TTAAGCCTGTGTCAACAGTTCTGA |
| H11-Positive | CGGAGGGCTTCTTATCTTAG | GTGTGGAGCTGTTTAGGGAC |
| 18s | GTAACCCGTTGAACCCCATT | CCATCCAATCGGTAGTAGCG |
| PNPLA3^I148M^ | AGGGTCTCTGCAAATGCCTC | ATATCGCACGCCTCTGAAGG |
| GIPR^dn^ | GTCGGAGATCCGCCGT | TCCTTGTAGTCGCAGTAACTTTC |
| hIAPP | CGGAAATGCAACACTGCCAC | GGCACGTCGTAGGGGTAAAG |
| SLA-4 | CTCTCCAGTGGACTTACGGC | GGTAGTTCCTATCGCGCTCC |
| SLA-6 | CCGCGGATGCCTGTTAGGAA | CTGACCCTTCTCCAGACGC |
| SLA-7 | CGTGGTGACTGGAGTTGTGA | CCCCCATAACCCTGTTGTGT |
| SLA-8 | CATCGTAGGAATCGTTGCTGGTCT | CATCAGAGTTCTCGACGCTGTTGTT |
| SLA-DRA | ATTGCTGTCCATCCTGACCC | ACTCGCCAGATTTGTCAGGG |
| IL15 | GGATGCAAAGAATGTGAGGAGC | CCTCTAAGCAGTGGCAAGATG |
| IL15RA | GTGCTCCTCCTTGTGGTACA | CATTGGCACGACTTCCATGC |
| IL2RG | ATAACCCGCTCTGTGGAAGC | TACACACACATGAGGCCGAC |
| CCL26 | CCACACGTGGCATTGATGTG | TTTGCACCCATGTTTCCTGC |
| CCL5 | ATATGCCTCGGACACCACAC | TGTACTCCCGCACCCATTTC |
| CD3E | GTGGATCTGATGGCAGTGGT | TCCGGATGGGCTCATAGTCT |
| CD40 | ACCACTTGTGTGTGCAGTGA | CTTTGCTCTCGCAGCTTGTC |
| CD40L | GACCCTGGAAAACGGGAGAC | GCTGGCTATGAAGGGAGCTT |
| CD8A | CCAGCCCTTCAGAGAGATTCA | CTGGGGAAACGGAAAGGAAAG |
| CD8B | ACTCCTTCGTCTGTGATGGC | GGAGTTGGAGAAGGTAGCGG |
| CTSW | GCCATCAAGCATCAGAAGGAC | GTCCCCTTGTACGGGTAGTC |
| IFNG | GCCATTCAAAGGAGCATGGA | TTCACTGATGGCTTTGCGCT |
| LCK | GCCTCTCCACTGCAAGACAA | CACTCGCCGTTCTGCTCTAA |
| ZAP70 | AGATCCCTGTCAGCAACGTG | CTGAGCGGGCAGTGTAGTAG |
| STAT4 | ATGCGGTGCTGAGAGAAAAC | CATTCCCATTTGGGGGTGTC |
| TBX21 | ATCATCACCAAGCAGGGACG | CGTCCACGAACATCCGGTAA |
| TSPO | ACGCAATGTCCTCGGAAAGG | TCATGTAGGAGCCATACCCCA |
| GPX3 | AACAGGAACCGGGAGACAAC | GTTCCAGCGGATGTCATGGA |
| GSTA4 | CCGCAGGAGTCGAGTTTGAT | TTGCCGAAGAGATGGTGCTT |
| VPS13D | ATCCTGGGCTCTGAGGAGTT | CCCAAGCGGACTAGAAGCAA |
| TTC36 | GCTTCAGCCTACAACAACCG | CTAGCATGCGGTTGCACAGA |
| LAYN | GACCTTTACGAGTGCTCCCC | GCCAGTTTTGTCCCTGCATC |
| NNAT | CTGCTCATCATCGGCTGGTA | TGAACACCTCACTTCTCGCA |
| RBP7 | CTGGTCAGCAGCGACAACTT | CTGCTGTTGGTGTGGATGGT |
| ISLR | TCTGCTCATCCCCGACTTTG | ATGGCTGCACCTCATTGTCA |
| COLGALT2 | CCGGATGTTACGCACACTCT | AGTCTTCTCCAGCTCTCGGT |
| SBSPON | GTACCTCCGAGAGGGCTACA | CCAAGTTCCCTGACACCGAG |
